# Precision luminosity measurement in proton–proton collisions at $\sqrt{s} = 13\,\hbox {TeV}$ in 2015 and 2016 at CMS

**DOI:** 10.1140/epjc/s10052-021-09538-2

**Published:** 2021-09-09

**Authors:** A. M. Sirunyan, A. Tumasyan, W. Adam, J. W. Andrejkovic, T. Bergauer, S. Chatterjee, M. Dragicevic, A. Escalante Del Valle, R. Frühwirth, M. Jeitler, N. Krammer, L. Lechner, D. Liko, I. Mikulec, F. M. Pitters, J. Schieck, R. Schöfbeck, M. Spanring, S. Templ, W. Waltenberger, C.-E. Wulz, V. Chekhovsky, A. Litomin, V. Makarenko, M. R. Darwish, E. A. De Wolf, X. Janssen, T. Kello, A. Lelek, H. Rejeb Sfar, P. Van Mechelen, S. Van Putte, N. Van Remortel, F. Blekman, E. S. Bols, J. D’Hondt, J. De Clercq, M. Delcourt, S. Lowette, S. Moortgat, A. Morton, D. Müller, A. R. Sahasransu, S. Tavernier, W. Van Doninck, P. Van Mulders, D. Beghin, B. Bilin, B. Clerbaux, G. De Lentdecker, L. Favart, A. Grebenyuk, A. K. Kalsi, K. Lee, M. Mahdavikhorrami, I. Makarenko, L. Moureaux, L. Pétré, A. Popov, N. Postiau, E. Starling, L. Thomas, M. Vanden Bemden, C. Vander Velde, P. Vanlaer, D. Vannerom, L. Wezenbeek, T. Cornelis, D. Dobur, M. Gruchala, G. Mestdach, M. Niedziela, C. Roskas, K. Skovpen, M. Tytgat, W. Verbeke, B. Vermassen, M. Vit, A. Bethani, G. Bruno, F. Bury, C. Caputo, P. David, C. Delaere, I. S. Donertas, A. Giammanco, V. Lemaitre, K. Mondal, J. Prisciandaro, A. Taliercio, M. Teklishyn, P. Vischia, S. Wertz, S. Wuyckens, G. A. Alves, C. Hensel, A. Moraes, W. L. Aldá Júnior, M. Barroso Ferreira Filho, H. Brandao Malbouisson, W. Carvalho, J. Chinellato, E. M. Da Costa, G. G. Da Silveira, D. De Jesus Damiao, S. Fonseca De Souza, D. Matos Figueiredo, C. Mora Herrera, K. Mota Amarilo, L. Mundim, H. Nogima, P. Rebello Teles, L. J. Sanchez Rosas, A. Santoro, S. M. Silva Do Amaral, A. Sznajder, M. Thiel, F. Torres Da Silva De Araujo, A. Vilela Pereira, C. A. Bernardes, L. Calligaris, T. R. Fernandez Perez Tomei, E. M. Gregores, D. S. Lemos, P. G. Mercadante, S. F. Novaes, Sandra S. Padula, A. Aleksandrov, G. Antchev, I. Atanasov, R. Hadjiiska, P. Iaydjiev, M. Misheva, M. Rodozov, M. Shopova, G. Sultanov, A. Dimitrov, T. Ivanov, L. Litov, B. Pavlov, P. Petkov, A. Petrov, T. Cheng, W. Fang, Q. Guo, T. Javaid, M. Mittal, H. Wang, L. Yuan, M. Ahmad, G. Bauer, C. Dozen, Z. Hu, J. Martins, Y. Wang, K. Yi, E. Chapon, G. M. Chen, H. S. Chen, M. Chen, A. Kapoor, D. Leggat, H. Liao, Z.-A. LIU, R. Sharma, A. Spiezia, J. Tao, J. Thomas-Wilsker, J. Wang, H. Zhang, S. Zhang, J. Zhao, A. Agapitos, Y. Ban, C. Chen, Q. Huang, A. Levin, Q. Li, M. Lu, X. Lyu, Y. Mao, S. J. Qian, D. Wang, Q. Wang, J. Xiao, Z. You, X. Gao, H. Okawa, M. Xiao, C. Avila, A. Cabrera, C. Florez, J. Fraga, A. Sarkar, M. A. Segura Delgado, J. Jaramillo, J. Mejia Guisao, F. Ramirez, J. D. Ruiz Alvarez, C. A. Salazar González, N. Vanegas Arbelaez, D. Giljanovic, N. Godinovic, D. Lelas, I. Puljak, Z. Antunovic, M. Kovac, T. Sculac, V. Brigljevic, D. Ferencek, D. Majumder, M. Roguljic, A. Starodumov, T. Susa, A. Attikis, E. Erodotou, A. Ioannou, G. Kole, M. Kolosova, S. Konstantinou, J. Mousa, C. Nicolaou, F. Ptochos, P. A. Razis, H. Rykaczewski, H. Saka, M. Finger, M. Finger Jr., A. Kveton, E. Ayala, E. Carrera Jarrin, S. Abu Zeid, S. Khalil, E. Salama, M. A. Mahmoud, Y. Mohammed, S. Bhowmik, A. Carvalho Antunes De Oliveira, R. K. Dewanjee, K. Ehataht, M. Kadastik, J. Pata, M. Raidal, C. Veelken, P. Eerola, L. Forthomme, H. Kirschenmann, K. Osterberg, M. Voutilainen, E. Brücken, F. Garcia, J. Havukainen, V. Karimäki, M. S. Kim, R. Kinnunen, T. Lampén, K. Lassila-Perini, S. Lehti, T. Lindén, H. Siikonen, E. Tuominen, J. Tuominiemi, P. Luukka, H. Petrow, T. Tuuva, C. Amendola, M. Besancon, F. Couderc, M. Dejardin, D. Denegri, J. L. Faure, F. Ferri, S. Ganjour, A. Givernaud, P. Gras, G. Hamel de Monchenault, P. Jarry, B. Lenzi, E. Locci, J. Malcles, J. Rander, A. Rosowsky, M. Ö. Sahin, A. Savoy-Navarro, M. Titov, G. B. Yu, S. Ahuja, F. Beaudette, M. Bonanomi, A. Buchot Perraguin, P. Busson, C. Charlot, O. Davignon, B. Diab, G. Falmagne, S. Ghosh, R. Granier de Cassagnac, A. Hakimi, I. Kucher, A. Lobanov, M. Nguyen, C. Ochando, P. Paganini, J. Rembser, R. Salerno, J. B. Sauvan, Y. Sirois, A. Zabi, A. Zghiche, J.-L. Agram, J. Andrea, D. Apparu, D. Bloch, G. Bourgatte, J.-M. Brom, E. C. Chabert, C. Collard, D. Darej, J.-C. Fontaine, U. Goerlach, C. Grimault, A.-C. Le Bihan, P. Van Hove, E. Asilar, S. Beauceron, C. Bernet, G. Boudoul, C. Camen, A. Carle, N. Chanon, D. Contardo, P. Depasse, H. El Mamouni, J. Fay, S. Gascon, M. Gouzevitch, B. Ille, Sa. Jain, I. B. Laktineh, H. Lattaud, A. Lesauvage, M. Lethuillier, L. Mirabito, K. Shchablo, L. Torterotot, G. Touquet, M. Vander Donckt, S. Viret, A. Khvedelidze, Z. Tsamalaidze, L. Feld, K. Klein, M. Lipinski, D. Meuser, A. Pauls, M. P. Rauch, J. Schulz, M. Teroerde, D. Eliseev, M. Erdmann, P. Fackeldey, B. Fischer, S. Ghosh, T. Hebbeker, K. Hoepfner, H. Keller, L. Mastrolorenzo, M. Merschmeyer, A. Meyer, G. Mocellin, S. Mondal, S. Mukherjee, D. Noll, A. Novak, T. Pook, A. Pozdnyakov, Y. Rath, H. Reithler, J. Roemer, A. Schmidt, S. C. Schuler, A. Sharma, S. Wiedenbeck, S. Zaleski, C. Dziwok, G. Flügge, W. Haj Ahmad, O. Hlushchenko, T. Kress, A. Nowack, C. Pistone, O. Pooth, D. Roy, H. Sert, A. Stahl, T. Ziemons, H. Aarup Petersen, M. Aldaya Martin, P. Asmuss, I. Babounikau, S. Baxter, O. Behnke, A. Bermúdez Martínez, A. A. Bin Anuar, K. Borras, V. Botta, D. Brunner, A. Campbell, A. Cardini, P. Connor, S. Consuegra Rodríguez, V. Danilov, M. M. Defranchis, L. Didukh, G. Eckerlin, D. Eckstein, L. I. Estevez Banos, E. Gallo, A. Geiser, A. Giraldi, A. Grohsjean, M. Guthoff, A. Harb, A. Jafari, N. Z. Jomhari, H. Jung, A. Kasem, M. Kasemann, H. Kaveh, C. Kleinwort, J. Knolle, D. Krücker, W. Lange, T. Lenz, J. Leonard, J. Lidrych, K. Lipka, W. Lohmann, T. Madlener, R. Mankel, I.-A. Melzer-Pellmann, J. Metwally, A. B. Meyer, M. Meyer, J. Mnich, A. Mussgiller, V. Myronenko, Y. Otarid, D. Pérez Adán, D. Pitzl, A. Raspereza, B. Ribeiro Lopes, J. Rübenach, A. Saggio, A. Saibel, M. Savitskyi, V. Scheurer, C. Schwanenberger, A. Singh, R. E. Sosa Ricardo, N. Tonon, O. Turkot, A. Vagnerini, M. Van De Klundert, R. Walsh, D. Walter, Y. Wen, K. Wichmann, C. Wissing, S. Wuchterl, R. Zlebcik, R. Aggleton, S. Bein, L. Benato, A. Benecke, K. De Leo, T. Dreyer, M. Eich, F. Feindt, A. Fröhlich, C. Garbers, E. Garutti, P. Gunnellini, J. Haller, A. Hinzmann, A. Karavdina, G. Kasieczka, R. Klanner, R. Kogler, V. Kutzner, J. Lange, T. Lange, A. Malara, A. Nigamova, K. J. Pena Rodriguez, O. Rieger, P. Schleper, M. Schröder, J. Schwandt, D. Schwarz, J. Sonneveld, H. Stadie, G. Steinbrück, A. Tews, B. Vormwald, I. Zoi, J. Bechtel, T. Berger, E. Butz, R. Caspart, T. Chwalek, W. De Boer, A. Dierlamm, A. Droll, K. El Morabit, N. Faltermann, K. Flöh, M. Giffels, J. O. Gosewisch, A. Gottmann, F. Hartmann, C. Heidecker, U. Husemann, I. Katkov, P. Keicher, R. Koppenhöfer, S. Maier, S. Mallows, M. Metzler, S. Mitra, Th. Müller, M. Musich, M. Neukum, G. Quast, K. Rabbertz, J. Rauser, D. Savoiu, D. Schäfer, M. Schnepf, D. Seith, I. Shvetsov, H. J. Simonis, R. Ulrich, J. Van Der Linden, R. F. Von Cube, M. Wassmer, M. Weber, S. Wieland, R. Wolf, S. Wozniewski, S. Wunsch, G. Anagnostou, P. Asenov, G. Daskalakis, T. Geralis, A. Kyriakis, D. Loukas, A. Stakia, M. Diamantopoulou, D. Karasavvas, G. Karathanasis, P. Kontaxakis, C. K. Koraka, A. Manousakis-Katsikakis, A. Panagiotou, I. Papavergou, N. Saoulidou, K. Theofilatos, E. Tziaferi, K. Vellidis, E. Vourliotis, G. Bakas, K. Kousouris, I. Papakrivopoulos, G. Tsipolitis, A. Zacharopoulou, I. Evangelou, C. Foudas, P. Gianneios, P. Katsoulis, P. Kokkas, N. Manthos, I. Papadopoulos, J. Strologas, M. Csanad, M. M. A. Gadallah, S. Lökös, P. Major, K. Mandal, A. Mehta, G. Pasztor, A. J. Rádl, O. Surányi, G. I. Veres, M. Bartók, G. Bencze, C. Hajdu, D. Horvath, F. Sikler, V. Veszpremi, G. Vesztergombi, S. Czellar, J. Karancsi, J. Molnar, Z. Szillasi, D. Teyssier, P. Raics, Z. L. Trocsanyi, B. Ujvari, T. Csorgo, F. Nemes, T. Novak, S. Choudhury, J. R. Komaragiri, D. Kumar, L. Panwar, P. C. Tiwari, S. Bahinipati, D. Dash, C. Kar, P. Mal, T. Mishra, V. K. Muraleedharan Nair Bindhu, A. Nayak, P. Saha, N. Sur, S. K. Swain, S. Bansal, S. B. Beri, V. Bhatnagar, G. Chaudhary, S. Chauhan, N. Dhingra, R. Gupta, A. Kaur, S. Kaur, P. Kumari, M. Meena, K. Sandeep, J. B. Singh, A. K. Virdi, A. Ahmed, A. Bhardwaj, B. C. Choudhary, R. B. Garg, M. Gola, S. Keshri, A. Kumar, M. Naimuddin, P. Priyanka, K. Ranjan, A. Shah, M. Bharti, R. Bhattacharya, S. Bhattacharya, D. Bhowmik, S. Dutta, B. Gomber, M. Maity, S. Nandan, P. Palit, P. K. Rout, G. Saha, B. Sahu, S. Sarkar, M. Sharan, B. Singh, S. Thakur, P. K. Behera, S. C. Behera, P. Kalbhor, A. Muhammad, R. Pradhan, P. R. Pujahari, A. Sharma, A. K. Sikdar, D. Dutta, V. Jha, V. Kumar, D. K. Mishra, K. Naskar, P. K. Netrakanti, L. M. Pant, P. Shukla, T. Aziz, S. Dugad, G. B. Mohanty, U. Sarkar, S. Banerjee, S. Bhattacharya, R. Chudasama, M. Guchait, S. Karmakar, S. Kumar, G. Majumder, K. Mazumdar, S. Mukherjee, D. Roy, S. Dube, B. Kansal, S. Pandey, A. Rane, A. Rastogi, S. Sharma, H. Bakhshiansohi, M. Zeinali, S. Chenarani, S. M. Etesami, M. Khakzad, M. Mohammadi Najafabadi, M. Felcini, M. Grunewald, M. Abbrescia, R. Aly, C. Aruta, A. Colaleo, D. Creanza, N. De Filippis, M. De Palma, A. Di Florio, A. Di Pilato, W. Elmetenawee, L. Fiore, A. Gelmi, M. Gul, G. Iaselli, M. Ince, S. Lezki, G. Maggi, M. Maggi, I. Margjeka, V. Mastrapasqua, J. A. Merlin, S. My, S. Nuzzo, A. Pompili, G. Pugliese, A. Ranieri, G. Selvaggi, L. Silvestris, F. M. Simone, R. Venditti, P. Verwilligen, G. Abbiendi, C. Battilana, D. Bonacorsi, L. Borgonovi, S. Braibant-Giacomelli, L. Brigliadori, R. Campanini, P. Capiluppi, A. Castro, F. R. Cavallo, C. Ciocca, M. Cuffiani, G. M. Dallavalle, T. Diotalevi, F. Fabbri, A. Fanfani, E. Fontanesi, P. Giacomelli, L. Giommi, C. Grandi, L. Guiducci, F. Iemmi, S. Lo Meo, S. Marcellini, G. Masetti, F. L. Navarria, A. Perrotta, F. Primavera, A. M. Rossi, T. Rovelli, G. P. Siroli, N. Tosi, S. Albergo, S. Costa, A. Di Mattia, R. Potenza, A. Tricomi, C. Tuve, G. Barbagli, A. Cassese, R. Ceccarelli, V. Ciulli, C. Civinini, R. D’Alessandro, F. Fiori, E. Focardi, G. Latino, P. Lenzi, M. Lizzo, M. Meschini, S. Paoletti, R. Seidita, G. Sguazzoni, L. Viliani, L. Benussi, S. Bianco, D. Piccolo, M. Bozzo, F. Ferro, R. Mulargia, E. Robutti, S. Tosi, A. Benaglia, F. Brivio, F. Cetorelli, V. Ciriolo, F. De Guio, M. E. Dinardo, P. Dini, S. Gennai, A. Ghezzi, P. Govoni, L. Guzzi, M. Malberti, S. Malvezzi, A. Massironi, D. Menasce, F. Monti, L. Moroni, M. Paganoni, D. Pedrini, S. Ragazzi, T. Tabarelli de Fatis, D. Valsecchi, D. Zuolo, S. Buontempo, F. Carnevali, N. Cavallo, A. De Iorio, F. Fabozzi, A. O. M. Iorio, L. Lista, S. Meola, P. Paolucci, B. Rossi, C. Sciacca, P. Azzi, N. Bacchetta, D. Bisello, P. Bortignon, A. Bragagnolo, R. Carlin, P. Checchia, P. De Castro Manzano, T. Dorigo, F. Gasparini, U. Gasparini, S. Y. Hoh, L. Layer, M. Margoni, A. T. Meneguzzo, M. Presilla, P. Ronchese, R. Rossin, F. Simonetto, G. Strong, M. Tosi, H. YARAR, M. Zanetti, P. Zotto, A. Zucchetta, G. Zumerle, C. Aime‘, A. Braghieri, S. Calzaferri, D. Fiorina, P. Montagna, S. P. Ratti, V. Re, M. Ressegotti, C. Riccardi, P. Salvini, I. Vai, P. Vitulo, G. M. Bilei, D. Ciangottini, L. Fanò, P. Lariccia, G. Mantovani, V. Mariani, M. Menichelli, F. Moscatelli, A. Piccinelli, A. Rossi, A. Santocchia, D. Spiga, T. Tedeschi, P. Azzurri, G. Bagliesi, V. Bertacchi, L. Bianchini, T. Boccali, E. Bossini, R. Castaldi, M. A. Ciocci, R. Dell’Orso, M. R. Di Domenico, S. Donato, A. Giassi, M. T. Grippo, F. Ligabue, E. Manca, G. Mandorli, A. Messineo, F. Palla, G. Ramirez-Sanchez, A. Rizzi, G. Rolandi, S. Roy Chowdhury, A. Scribano, N. Shafiei, P. Spagnolo, R. Tenchini, G. Tonelli, N. Turini, A. Venturi, P. G. Verdini, F. Cavallari, M. Cipriani, D. Del Re, E. Di Marco, M. Diemoz, E. Longo, P. Meridiani, G. Organtini, F. Pandolfi, R. Paramatti, C. Quaranta, S. Rahatlou, C. Rovelli, F. Santanastasio, L. Soffi, R. Tramontano, N. Amapane, R. Arcidiacono, S. Argiro, M. Arneodo, N. Bartosik, R. Bellan, A. Bellora, J. Berenguer Antequera, C. Biino, A. Cappati, N. Cartiglia, S. Cometti, M. Costa, R. Covarelli, N. Demaria, B. Kiani, F. Legger, C. Mariotti, S. Maselli, E. Migliore, V. Monaco, E. Monteil, M. Monteno, M. M. Obertino, G. Ortona, L. Pacher, N. Pastrone, M. Pelliccioni, G. L. Pinna Angioni, M. Ruspa, R. Salvatico, K. Shchelina, F. Siviero, V. Sola, A. Solano, D. Soldi, A. Staiano, M. Tornago, D. Trocino, S. Belforte, V. Candelise, M. Casarsa, F. Cossutti, A. Da Rold, G. Della Ricca, G. Sorrentino, F. Vazzoler, S. Dogra, C. Huh, B. Kim, D. H. Kim, G. N. Kim, J. Lee, S. W. Lee, C. S. Moon, Y. D. Oh, S. I. Pak, B. C. Radburn-Smith, S. Sekmen, Y. C. Yang, H. Kim, D. H. Moon, T. J. Kim, J. Park, S. Cho, S. Choi, Y. Go, B. Hong, K. Lee, K. S. Lee, J. Lim, J. Park, S. K. Park, J. Yoo, J. Goh, A. Gurtu, H. S. Kim, Y. Kim, J. Almond, J. H. Bhyun, J. Choi, S. Jeon, J. Kim, J. S. Kim, S. Ko, H. Kwon, H. Lee, S. Lee, B. H. Oh, M. Oh, S. B. Oh, H. Seo, U. K. Yang, I. Yoon, D. Jeon, J. H. Kim, B. Ko, J. S. H. Lee, I. C. Park, Y. Roh, D. Song, I. J. Watson, S. Ha, H. D. Yoo, Y. Choi, Y. Jeong, H. Lee, Y. Lee, I. Yu, T. Beyrouthy, Y. Maghrbi, V. Veckalns, M. Ambrozas, A. Juodagalvis, A. Rinkevicius, G. Tamulaitis, A. Vaitkevicius, W. A. T. Wan Abdullah, M. N. Yusli, Z. Zolkapli, J. F. Benitez, A. Castaneda Hernandez, J. A. Murillo Quijada, L. Valencia Palomo, G. Ayala, H. Castilla-Valdez, E. De La Cruz-Burelo, I. Heredia-De La Cruz, R. Lopez-Fernandez, C. A. Mondragon Herrera, D. A. Perez Navarro, A. Sanchez-Hernandez, S. Carrillo Moreno, C. Oropeza Barrera, M. Ramirez-Garcia, F. Vazquez Valencia, I. Pedraza, H. A. Salazar Ibarguen, C. Uribe Estrada, J. Mijuskovic, N. Raicevic, D. Krofcheck, S. Bheesette, A. P. H. Butler, P. H. Butler, A. Lokhovitskiy, P. Lujan, A. Ahmad, M. I. Asghar, A. Awais, M. I. M. Awan, H. R. Hoorani, W. A. Khan, M. A. Shah, M. Shoaib, M. Waqas, V. Avati, L. Grzanka, M. Malawski, H. Bialkowska, M. Bluj, B. Boimska, T. Frueboes, M. Górski, M. Kazana, M. Szleper, P. Traczyk, P. Zalewski, K. Bunkowski, K. Doroba, A. Kalinowski, M. Konecki, J. Krolikowski, M. Walczak, M. Araujo, P. Bargassa, D. Bastos, A. Boletti, P. Faccioli, M. Gallinaro, J. Hollar, N. Leonardo, T. Niknejad, J. Seixas, O. Toldaiev, J. Varela, S. Afanasiev, D. Budkouski, P. Bunin, M. Gavrilenko, I. Golutvin, I. Gorbunov, A. Kamenev, V. Karjavine, A. Lanev, A. Malakhov, V. Matveev, V. Palichik, V. Perelygin, M. Savina, D. Seitova, V. Shalaev, S. Shmatov, S. Shulha, V. Smirnov, O. Teryaev, N. Voytishin, A. Zarubin, I. Zhizhin, G. Gavrilov, V. Golovtcov, Y. Ivanov, V. Kim, E. Kuznetsova, V. Murzin, V. Oreshkin, I. Smirnov, D. Sosnov, V. Sulimov, L. Uvarov, S. Volkov, A. Vorobyev, Yu. Andreev, A. Dermenev, S. Gninenko, N. Golubev, A. Karneyeu, M. Kirsanov, N. Krasnikov, A. Pashenkov, G. Pivovarov, D. Tlisov, A. Toropin, V. Epshteyn, V. Gavrilov, N. Lychkovskaya, A. Nikitenko, V. Popov, G. Safronov, A. Spiridonov, A. Stepennov, M. Toms, E. Vlasov, A. Zhokin, T. Aushev, O. Bychkova, M. Danilov, P. Parygin, E. Popova, V. Rusinov, V. Andreev, M. Azarkin, I. Dremin, M. Kirakosyan, A. Terkulov, A. Belyaev, E. Boos, M. Dubinin, L. Dudko, A. Ershov, A. Gribushin, A. Kaminskiy, V. Klyukhin, O. Kodolova, I. Lokhtin, S. Obraztsov, S. Petrushanko, V. Savrin, V. Blinov, T. Dimova, L. Kardapoltsev, I. Ovtin, Y. Skovpen, I. Azhgirey, I. Bayshev, V. Kachanov, A. Kalinin, D. Konstantinov, V. Petrov, R. Ryutin, A. Sobol, S. Troshin, N. Tyurin, A. Uzunian, A. Volkov, A. Babaev, V. Okhotnikov, L. Sukhikh, V. Borchsh, V. Ivanchenko, E. Tcherniaev, P. Adzic, M. Dordevic, P. Milenovic, J. Milosevic, V. Milosevic, M. Aguilar-Benitez, J. Alcaraz Maestre, A. Álvarez Fernández, I. Bachiller, M. Barrio Luna, Cristina F. Bedoya, C. A. Carrillo Montoya, M. Cepeda, M. Cerrada, N. Colino, B. De La Cruz, A. Delgado Peris, J. P. Fernández Ramos, J. Flix, M. C. Fouz, O. Gonzalez Lopez, S. Goy Lopez, J. M. Hernandez, M. I. Josa, J. León Holgado, D. Moran, Á. Navarro Tobar, A. Pérez-Calero Yzquierdo, J. Puerta Pelayo, I. Redondo, L. Romero, S. Sánchez Navas, M. S. Soares, L. Urda Gómez, C. Willmott, J. F. de Trocóniz, R. Reyes-Almanza, B. Alvarez Gonzalez, J. Cuevas, C. Erice, J. Fernandez Menendez, S. Folgueras, I. Gonzalez Caballero, E. Palencia Cortezon, C. Ramón Álvarez, J. Ripoll Sau, V. Rodríguez Bouza, A. Trapote, J. A. Brochero Cifuentes, I. J. Cabrillo, A. Calderon, B. Chazin Quero, J. Duarte Campderros, M. Fernandez, C. Fernandez Madrazo, P. J. Fernández Manteca, A. García Alonso, G. Gomez, C. Martinez Rivero, P. Martinez Ruiz del Arbol, F. Matorras, J. Piedra Gomez, C. Prieels, F. Ricci-Tam, T. Rodrigo, A. Ruiz-Jimeno, L. Scodellaro, N. Trevisani, I. Vila, J. M. Vizan Garcia, M. K. Jayananda, B. Kailasapathy, D. U. J. Sonnadara, D. D. C. Wickramarathna, W. G. D. Dharmaratna, K. Liyanage, N. Perera, N. Wickramage, T. K. Aarrestad, D. Abbaneo, J. Alimena, E. Auffray, G. Auzinger, J. Baechler, P. Baillon, A. H. Ball, D. Barney, J. Bendavid, N. Beni, M. Bianco, A. Bocci, E. Brondolin, T. Camporesi, M. Capeans Garrido, G. Cerminara, S. S. Chhibra, L. Cristella, D. d’Enterria, A. Dabrowski, N. Daci, A. David, A. De Roeck, M. Deile, R. Di Maria, M. Dobson, M. Dünser, N. Dupont, A. Elliott-Peisert, N. Emriskova, F. Fallavollita, D. Fasanella, S. Fiorendi, A. Florent, G. Franzoni, J. Fulcher, W. Funk, S. Giani, D. Gigi, K. Gill, F. Glege, L. Gouskos, M. Haranko, J. Hegeman, Y. Iiyama, V. Innocente, T. James, P. Janot, J. Kaspar, J. Kieseler, M. Komm, N. Kratochwil, C. Lange, S. Laurila, P. Lecoq, K. Long, C. Lourenço, L. Malgeri, S. Mallios, M. Mannelli, F. Meijers, S. Mersi, E. Meschi, F. Moortgat, M. Mulders, S. Orfanelli, L. Orsini, F. Pantaleo, L. Pape, E. Perez, M. Peruzzi, A. Petrilli, G. Petrucciani, A. Pfeiffer, M. Pierini, H. Qu, T. Quast, D. Rabady, A. Racz, M. Rieger, M. Rovere, H. Sakulin, J. Salfeld-Nebgen, S. Scarfi, C. Schäfer, C. Schwick, M. Selvaggi, A. Sharma, P. Silva, W. Snoeys, P. Sphicas, S. Summers, V. R. Tavolaro, D. Treille, A. Tsirou, P. Tsrunchev, G. P. Van Onsem, M. Verzetti, J. Wanczyk, K. A. Wozniak, W. D. Zeuner, L. Caminada, A. Ebrahimi, W. Erdmann, R. Horisberger, Q. Ingram, H. C. Kaestli, D. Kotlinski, U. Langenegger, M. Missiroli, T. Rohe, K. Androsov, M. Backhaus, P. Berger, A. Calandri, N. Chernyavskaya, A. De Cosa, G. Dissertori, M. Dittmar, M. Donegà, C. Dorfer, F. Eble, T. Gadek, T. A. Gómez Espinosa, C. Grab, D. Hits, W. Lustermann, A.-M. Lyon, R. A. Manzoni, C. Martin Perez, M. T. Meinhard, F. Micheli, F. Nessi-Tedaldi, J. Niedziela, F. Pauss, V. Perovic, G. Perrin, S. Pigazzini, M. G. Ratti, M. Reichmann, C. Reissel, T. Reitenspiess, B. Ristic, D. Ruini, D. A. Sanz Becerra, M. Schönenberger, V. Stampf, J. Steggemann, R. Wallny, D. H. Zhu, C. Amsler, C. Botta, D. Brzhechko, M. F. Canelli, A. De Wit, R. Del Burgo, J. K. Heikkilä, M. Huwiler, A. Jofrehei, B. Kilminster, S. Leontsinis, A. Macchiolo, P. Meiring, V. M. Mikuni, U. Molinatti, I. Neutelings, G. Rauco, A. Reimers, P. Robmann, S. Sanchez Cruz, K. Schweiger, Y. Takahashi, C. Adloff, C. M. Kuo, W. Lin, A. Roy, T. Sarkar, S. S. Yu, L. Ceard, P. Chang, Y. Chao, K. F. Chen, P. H. Chen, W.-S. Hou, Y. y. Li, R.-S. Lu, E. Paganis, A. Psallidas, A. Steen, E. Yazgan, P. r. Yu, B. Asavapibhop, C. Asawatangtrakuldee, N. Srimanobhas, F. Boran, S. Damarseckin, Z. S. Demiroglu, F. Dolek, I. Dumanoglu, E. Eskut, G. Gokbulut, Y. Guler, E. Gurpinar Guler, I. Hos, C. Isik, E. E. Kangal, O. Kara, A. Kayis Topaksu, U. Kiminsu, G. Onengut, K. Ozdemir, A. Polatoz, A. E. Simsek, B. Tali, U. G. Tok, S. Turkcapar, I. S. Zorbakir, C. Zorbilmez, B. Isildak, G. Karapinar, K. Ocalan, M. Yalvac, B. Akgun, I. O. Atakisi, Y. C. Cekmecelioglu, E. Gülmez, M. Kaya, O. Kaya, Ö. Özçelik, S. Tekten, E. A. Yetkin, A. Cakir, K. Cankocak, Y. Komurcu, S. Sen, F. Aydogmus Sen, S. Cerci, B. Kaynak, S. Ozkorucuklu, D. Sunar Cerci, B. Grynyov, L. Levchuk, E. Bhal, S. Bologna, J. J. Brooke, A. Bundock, E. Clement, D. Cussans, H. Flacher, J. Goldstein, G. P. Heath, H. F. Heath, L. Kreczko, B. Krikler, S. Paramesvaran, T. Sakuma, S. Seif El Nasr-Storey, V. J. Smith, N. Stylianou, J. Taylor, A. Titterton, K. W. Bell, A. Belyaev, C. Brew, R. M. Brown, D. J. A. Cockerill, K. V. Ellis, K. Harder, S. Harper, J. Linacre, K. Manolopoulos, D. M. Newbold, E. Olaiya, D. Petyt, T. Reis, T. Schuh, C. H. Shepherd-Themistocleous, A. Thea, I. R. Tomalin, T. Williams, R. Bainbridge, P. Bloch, S. Bonomally, J. Borg, S. Breeze, O. Buchmuller, V. Cepaitis, G. S. Chahal, D. Colling, P. Dauncey, G. Davies, M. Della Negra, S. Fayer, G. Fedi, G. Hall, M. H. Hassanshahi, G. Iles, J. Langford, L. Lyons, A.-M. Magnan, S. Malik, A. Martelli, J. Nash, V. Palladino, M. Pesaresi, D. M. Raymond, A. Richards, A. Rose, E. Scott, C. Seez, A. Shtipliyski, A. Tapper, K. Uchida, T. Virdee, N. Wardle, S. N. Webb, D. Winterbottom, A. G. Zecchinelli, J. E. Cole, A. Khan, P. Kyberd, C. K. Mackay, I. D. Reid, L. Teodorescu, S. Zahid, S. Abdullin, A. Brinkerhoff, B. Caraway, J. Dittmann, K. Hatakeyama, A. R. Kanuganti, B. McMaster, N. Pastika, S. Sawant, C. Smith, C. Sutantawibul, J. Wilson, R. Bartek, A. Dominguez, R. Uniyal, A. M. Vargas Hernandez, A. Buccilli, O. Charaf, S. I. Cooper, D. Di Croce, S. V. Gleyzer, C. Henderson, C. U. Perez, P. Rumerio, C. West, A. Akpinar, A. Albert, D. Arcaro, C. Cosby, Z. Demiragli, D. Gastler, J. Rohlf, K. Salyer, D. Sperka, D. Spitzbart, I. Suarez, A. Tsatsos, S. Yuan, D. Zou, G. Benelli, B. Burkle, X. Coubez, D. Cutts, Y. t. Duh, M. Hadley, U. Heintz, J. M. Hogan, E. Laird, G. Landsberg, K. T. Lau, J. Lee, J. Luo, M. Narain, S. Sagir, E. Usai, W. Y. Wong, X. Yan, D. Yu, W. Zhang, C. Brainerd, R. Breedon, M. Calderon De La Barca Sanchez, M. Chertok, J. Conway, P. T. Cox, R. Erbacher, F. Jensen, O. Kukral, R. Lander, M. Mulhearn, D. Pellett, B. Regnery, D. Taylor, M. Tripathi, Y. Yao, F. Zhang, M. Bachtis, R. Cousins, A. Dasgupta, A. Datta, D. Hamilton, J. Hauser, M. Ignatenko, M. A. Iqbal, T. Lam, N. Mccoll, W. A. Nash, S. Regnard, D. Saltzberg, C. Schnaible, B. Stone, V. Valuev, K. Burt, Y. Chen, R. Clare, J. W. Gary, G. Hanson, G. Karapostoli, O. R. Long, N. Manganelli, M. Olmedo Negrete, W. Si, S. Wimpenny, Y. Zhang, J. G. Branson, P. Chang, S. Cittolin, S. Cooperstein, N. Deelen, J. Duarte, R. Gerosa, L. Giannini, D. Gilbert, J. Guiang, R. Kansal, V. Krutelyov, R. Lee, J. Letts, M. Masciovecchio, S. May, S. Padhi, M. Pieri, B. V. Sathia Narayanan, V. Sharma, M. Tadel, A. Vartak, F. Würthwein, Y. Xiang, A. Yagil, N. Amin, C. Campagnari, M. Citron, A. Dorsett, V. Dutta, J. Incandela, M. Kilpatrick, B. Marsh, H. Mei, A. Ovcharova, M. Quinnan, J. Richman, U. Sarica, D. Stuart, S. Wang, A. Bornheim, O. Cerri, I. Dutta, J. M. Lawhorn, N. Lu, J. Mao, H. B. Newman, J. Ngadiuba, T. Q. Nguyen, M. Spiropulu, J. R. Vlimant, C. Wang, S. Xie, Z. Zhang, R. Y. Zhu, J. Alison, M. B. Andrews, T. Ferguson, T. Mudholkar, M. Paulini, I. Vorobiev, J. P. Cumalat, W. T. Ford, E. MacDonald, R. Patel, A. Perloff, K. Stenson, K. A. Ulmer, S. R. Wagner, J. Alexander, Y. Cheng, J. Chu, D. J. Cranshaw, K. Mcdermott, J. Monroy, J. R. Patterson, D. Quach, J. Reichert, A. Ryd, W. Sun, S. M. Tan, Z. Tao, J. Thom, P. Wittich, M. Zientek, M. Albrow, M. Alyari, G. Apollinari, A. Apresyan, A. Apyan, S. Banerjee, L. A. T. Bauerdick, A. Beretvas, D. Berry, J. Berryhill, P. C. Bhat, K. Burkett, J. N. Butler, A. Canepa, G. B. Cerati, H. W. K. Cheung, F. Chlebana, M. Cremonesi, K. F. Di Petrillo, V. D. Elvira, J. Freeman, Z. Gecse, L. Gray, D. Green, S. Grünendahl, O. Gutsche, R. M. Harris, R. Heller, T. C. Herwig, J. Hirschauer, B. Jayatilaka, S. Jindariani, M. Johnson, U. Joshi, P. Klabbers, T. Klijnsma, B. Klima, M. J. Kortelainen, K. H. M. Kwok, S. Lammel, D. Lincoln, R. Lipton, T. Liu, J. Lykken, C. Madrid, K. Maeshima, C. Mantilla, D. Mason, P. McBride, P. Merkel, S. Mrenna, S. Nahn, V. O’Dell, V. Papadimitriou, K. Pedro, C. Pena, O. Prokofyev, F. Ravera, A. Reinsvold Hall, L. Ristori, B. Schneider, E. Sexton-Kennedy, N. Smith, A. Soha, L. Spiegel, S. Stoynev, J. Strait, L. Taylor, S. Tkaczyk, N. V. Tran, L. Uplegger, E. W. Vaandering, H. A. Weber, A. Woodard, D. Acosta, P. Avery, D. Bourilkov, L. Cadamuro, V. Cherepanov, F. Errico, R. D. Field, D. Guerrero, B. M. Joshi, M. Kim, J. Konigsberg, A. Korytov, K. H. Lo, K. Matchev, N. Menendez, G. Mitselmakher, D. Rosenzweig, K. Shi, J. Sturdy, J. Wang, E. Yigitbasi, X. Zuo, T. Adams, A. Askew, D. Diaz, R. Habibullah, S. Hagopian, V. Hagopian, K. F. Johnson, R. Khurana, T. Kolberg, G. Martinez, H. Prosper, C. Schiber, R. Yohay, J. Zhang, M. M. Baarmand, S. Butalla, T. Elkafrawy, M. Hohlmann, R. Kumar Verma, D. Noonan, M. Rahmani, M. Saunders, F. Yumiceva, M. R. Adams, L. Apanasevich, H. Becerril Gonzalez, R. Cavanaugh, X. Chen, S. Dittmer, O. Evdokimov, C. E. Gerber, D. A. Hangal, D. J. Hofman, C. Mills, G. Oh, T. Roy, M. B. Tonjes, N. Varelas, J. Viinikainen, X. Wang, Z. Wu, Z. Ye, M. Alhusseini, K. Dilsiz, S. Durgut, R. P. Gandrajula, M. Haytmyradov, V. Khristenko, O. K. Köseyan, J.-P. Merlo, A. Mestvirishvili, A. Moeller, J. Nachtman, H. Ogul, Y. Onel, F. Ozok, A. Penzo, C. Snyder, E. Tiras, J. Wetzel, O. Amram, B. Blumenfeld, L. Corcodilos, M. Eminizer, A. V. Gritsan, S. Kyriacou, P. Maksimovic, J. Roskes, M. Swartz, T.Á. Vámi, C. Baldenegro Barrera, P. Baringer, A. Bean, A. Bylinkin, T. Isidori, S. Khalil, J. King, G. Krintiras, A. Kropivnitskaya, C. Lindsey, N. Minafra, M. Murray, C. Rogan, C. Royon, S. Sanders, E. Schmitz, J. D. Tapia Takaki, Q. Wang, J. Williams, G. Wilson, S. Duric, A. Ivanov, K. Kaadze, D. Kim, Y. Maravin, T. Mitchell, A. Modak, K. Nam, F. Rebassoo, D. Wright, E. Adams, A. Baden, O. Baron, A. Belloni, S. C. Eno, Y. Feng, N. J. Hadley, S. Jabeen, R. G. Kellogg, T. Koeth, A. C. Mignerey, S. Nabili, M. Seidel, A. Skuja, S. C. Tonwar, L. Wang, K. Wong, D. Abercrombie, G. Andreassi, R. Bi, S. Brandt, W. Busza, I. A. Cali, Y. Chen, M. D’Alfonso, G. Gomez Ceballos, M. Goncharov, P. Harris, M. Hu, M. Klute, D. Kovalskyi, J. Krupa, Y.-J. Lee, B. Maier, A. C. Marini, C. Mironov, C. Paus, D. Rankin, C. Roland, G. Roland, Z. Shi, G. S. F. Stephans, K. Tatar, J. Wang, Z. Wang, B. Wyslouch, R. M. Chatterjee, A. Evans, P. Hansen, J. Hiltbrand, Sh. Jain, M. Krohn, Y. Kubota, Z. Lesko, J. Mans, M. Revering, R. Rusack, R. Saradhy, N. Schroeder, N. Strobbe, M. A. Wadud, J. G. Acosta, S. Oliveros, K. Bloom, M. Bryson, S. Chauhan, D. R. Claes, C. Fangmeier, L. Finco, F. Golf, J. R. González Fernández, C. Joo, I. Kravchenko, J. E. Siado, G. R. Snow, W. Tabb, F. Yan, G. Agarwal, H. Bandyopadhyay, L. Hay, I. Iashvili, A. Kharchilava, C. McLean, D. Nguyen, J. Pekkanen, S. Rappoccio, A. Williams, G. Alverson, E. Barberis, C. Freer, Y. Haddad, A. Hortiangtham, J. Li, G. Madigan, B. Marzocchi, D. M. Morse, V. Nguyen, T. Orimoto, A. Parker, L. Skinnari, A. Tishelman-Charny, T. Wamorkar, B. Wang, A. Wisecarver, D. Wood, S. Bhattacharya, J. Bueghly, Z. Chen, A. Gilbert, T. Gunter, K. A. Hahn, N. Odell, M. H. Schmitt, K. Sung, M. Velasco, R. Band, R. Bucci, N. Dev, R. Goldouzian, M. Hildreth, K. Hurtado Anampa, C. Jessop, K. Lannon, N. Loukas, N. Marinelli, I. Mcalister, F. Meng, K. Mohrman, Y. Musienko, R. Ruchti, P. Siddireddy, M. Wayne, A. Wightman, M. Wolf, M. Zarucki, L. Zygala, B. Bylsma, B. Cardwell, L. S. Durkin, B. Francis, C. Hill, A. Lefeld, B. L. Winer, B. R. Yates, F. M. Addesa, B. Bonham, P. Das, G. Dezoort, P. Elmer, A. Frankenthal, B. Greenberg, N. Haubrich, S. Higginbotham, A. Kalogeropoulos, G. Kopp, S. Kwan, D. Lange, M. T. Lucchini, D. Marlow, K. Mei, I. Ojalvo, J. Olsen, C. Palmer, D. Stickland, C. Tully, Z. Xie, S. Malik, S. Norberg, A. S. Bakshi, V. E. Barnes, R. Chawla, S. Das, L. Gutay, M. Jones, A. W. Jung, S. Karmarkar, M. Liu, G. Negro, N. Neumeister, G. Paspalaki, C. C. Peng, S. Piperov, A. Purohit, J. F. Schulte, M. Stojanovic, J. Thieman, F. Wang, R. Xiao, W. Xie, J. Dolen, N. Parashar, A. Baty, S. Dildick, K. M. Ecklund, S. Freed, F. J. M. Geurts, A. Kumar, W. Li, B. P. Padley, R. Redjimi, J. Roberts, W. Shi, A. G. Stahl Leiton, A. Bodek, P. de Barbaro, R. Demina, J. L. Dulemba, C. Fallon, T. Ferbel, M. Galanti, A. Garcia-Bellido, O. Hindrichs, A. Khukhunaishvili, E. Ranken, R. Taus, B. Chiarito, J. P. Chou, A. Gandrakota, Y. Gershtein, E. Halkiadakis, A. Hart, M. Heindl, E. Hughes, S. Kaplan, O. Karacheban, I. Laflotte, A. Lath, R. Montalvo, K. Nash, M. Osherson, S. Salur, S. Schnetzer, S. Somalwar, R. Stone, S. A. Thayil, S. Thomas, H. Wang, H. Acharya, A. G. Delannoy, S. Spanier, O. Bouhali, M. Dalchenko, A. Delgado, R. Eusebi, J. Gilmore, T. Huang, T. Kamon, H. Kim, S. Luo, S. Malhotra, R. Mueller, D. Overton, D. Rathjens, A. Safonov, N. Akchurin, J. Damgov, V. Hegde, S. Kunori, K. Lamichhane, S. W. Lee, T. Mengke, S. Muthumuni, T. Peltola, S. Undleeb, I. Volobouev, Z. Wang, A. Whitbeck, E. Appelt, S. Greene, A. Gurrola, W. Johns, C. Maguire, A. Melo, H. Ni, K. Padeken, F. Romeo, P. Sheldon, S. Tuo, J. Velkovska, M. W. Arenton, B. Cox, G. Cummings, J. Hakala, R. Hirosky, M. Joyce, A. Ledovskoy, A. Li, C. Neu, B. Tannenwald, E. Wolfe, P. E. Karchin, N. Poudyal, P. Thapa, K. Black, T. Bose, J. Buchanan, C. Caillol, S. Dasu, I. De Bruyn, P. Everaerts, F. Fienga, C. Galloni, H. He, M. Herndon, A. Hervé, U. Hussain, A. Lanaro, A. Loeliger, R. Loveless, J. Madhusudanan Sreekala, A. Mallampalli, A. Mohammadi, D. Pinna, A. Savin, V. Shang, V. Sharma, W. H. Smith, D. Teague, S. Trembath-Reichert, W. Vetens, CMS Collaboration

**Affiliations:** 1 Yerevan Physics Institute, Yerevan, Armenia; 2 Institut für Hochenergiephysik, Vienna, Austria; 3 Institute for Nuclear Problems, Minsk, Belarus; 4 Universiteit Antwerpen, Antwerp, Belgium; 5 Vrije Universiteit Brussel, Brussels, Belgium; 6 Université Libre de Bruxelles, Brussels, Belgium; 7 Ghent University, Ghent, Belgium; 8 Université Catholique de Louvain, Louvain-la-Neuve, Belgium; 9 Centro Brasileiro de Pesquisas Fisicas, Rio de Janeiro, Brazil; 10 Universidade do Estado do Rio de Janeiro, Rio de Janeiro, Brazil; 11 Universidade Estadual Paulista, Universidade Federal do ABC, São Paulo, Brazil; 12 Institute for Nuclear Research and Nuclear Energy, Bulgarian Academy of Sciences, Sofia, Bulgaria; 13 University of Sofia, Sofia, Bulgaria; 14 Beihang University, Beijing, China; 15 Department of Physics, Tsinghua University, Beijing, China; 16 Institute of High Energy Physics, Beijing, China; 17 State Key Laboratory of Nuclear Physics and Technology, Peking University, Beijing, China; 18 Sun Yat-Sen University, Guangzhou, China; 19 Institute of Modern Physics and Key Laboratory of Nuclear Physics and Ion-beam Application (MOE)-Fudan University, Shanghai, China; 20 Zhejiang University, Hangzhou, China; 21 Universidad de Los Andes, Bogotá, Colombia; 22 Universidad de Antioquia, Medellín, Colombia; 23 Faculty of Electrical Engineering, Mechanical Engineering and Naval Architecture, University of Split, Split, Croatia; 24 Faculty of Science, University of Split, Split, Croatia; 25 Institute Rudjer Boskovic, Zagreb, Croatia; 26 University of Cyprus, Nicosia, Cyprus; 27 Charles University, Prague, Czech Republic; 28 Escuela Politecnica Nacional, Quito, Ecuador; 29 Universidad San Francisco de Quito, Quito, Ecuador; 30 Academy of Scientific Research and Technology of the Arab Republic of Egypt, Egyptian Network of High Energy Physics, Cairo, Egypt; 31 Center for High Energy Physics (CHEP-FU), Fayoum University, El-Fayoum, Egypt; 32 National Institute of Chemical Physics and Biophysics, Tallinn, Estonia; 33 Department of Physics, University of Helsinki, Helsinki, Finland; 34 Helsinki Institute of Physics, Helsinki, Finland; 35 Lappeenranta University of Technology, Lappeenranta, Finland; 36 IRFU, CEA, Université Paris-Saclay, Gif-sur-Yvette, France; 37 Laboratoire Leprince-Ringuet, CNRS/IN2P3, Ecole Polytechnique, Institut Polytechnique de Paris, Palaiseau, France; 38 Université de Strasbourg, CNRS, IPHC UMR 7178, Strasbourg, France; 39 Institut de Physique des 2 Infinis de Lyon (IP2I ), Villeurbanne, France; 40 Georgian Technical University, Tbilisi, Georgia; 41 I. Physikalisches Institut, RWTH Aachen University, Aachen, Germany; 42 III. Physikalisches Institut A, RWTH Aachen University, Aachen, Germany; 43 III. Physikalisches Institut B, RWTH Aachen University, Aachen, Germany; 44 Deutsches Elektronen-Synchrotron, Hamburg, Germany; 45 University of Hamburg, Hamburg, Germany; 46 Karlsruher Institut fuer Technologie, Karlsruhe, Germany; 47 Institute of Nuclear and Particle Physics (INPP), NCSR Demokritos, Aghia Paraskevi, Greece; 48 National and Kapodistrian University of Athens, Athens, Greece; 49 National Technical University of Athens, Athens, Greece; 50 University of Ioánnina, Ioannina, Greece; 51 MTA-ELTE Lendület CMS Particle and Nuclear Physics Group, Eötvös Loránd University, Budapest, Hungary; 52 Wigner Research Centre for Physics, Budapest, Hungary; 53 Institute of Nuclear Research ATOMKI, Debrecen, Hungary; 54 Institute of Physics, University of Debrecen, Debrecen, Hungary; 55 Eszterhazy Karoly University, Karoly Robert Campus, Gyongyos, Hungary; 56 Indian Institute of Science (IISc), Bangalore, India; 57 National Institute of Science Education and Research, HBNI, Bhubaneswar, India; 58 Panjab University, Chandigarh, India; 59 University of Delhi, Delhi, India; 60 Saha Institute of Nuclear Physics, HBNI, Kolkata, India; 61 Indian Institute of Technology Madras, Chennai, India; 62 Bhabha Atomic Research Centre, Mumbai, India; 63 Tata Institute of Fundamental Research-A, Mumbai, India; 64 Tata Institute of Fundamental Research-B, Mumbai, India; 65 Indian Institute of Science Education and Research (IISER), Pune, India; 66 Department of Physics, Isfahan University of Technology, Isfahan, Iran; 67 Institute for Research in Fundamental Sciences (IPM), Tehran, Iran; 68 University College Dublin, Dublin, Ireland; 69 INFN Sezione di Bari, Universit’a di Bari, Politecnico di Bari, Bari, Italy; 70 INFN Sezione di Bologna, Università di Bologna, Bologna, Italy; 71 INFN Sezione di Catania, Università di Catania, Catania, Italy; 72 INFN Sezione di Firenze, Università di Firenze, Florence, Italy; 73 INFN Laboratori Nazionali di Frascati, Frascati, Italy; 74 INFN Sezione di Genova, Università di Genova, Genoa, Italy; 75 INFN Sezione di Milano-Bicocca, Università di Milano-Bicocca, Milan, Italy; 76 INFN Sezione di Napoli, Università di Napoli ‘Federico II’, Naples, Italy, Università della Basilicata, Potenza, Italy, Università G. Marconi, Rome, Italy; 77 INFN Sezione di Padova, Università di Padova, Padua, Italy, Università di Trento, Trento, Italy; 78 INFN Sezione di Pavia, Università di Pavia, Pavia, Italy; 79 INFN Sezione di Perugia, Università di Perugia, Perugia, Italy; 80 INFN Sezione di Pisa, Università di Pisa, Scuola Normale Superiore di Pisa, Pisa, Italy, Università di Siena, Siena, Italy; 81 INFN Sezione di Roma, Sapienza Università di Roma, Rome, Italy; 82 INFN Sezione di Torino, Università di Torino, Turin, Italy, Università del Piemonte Orientale, Novara, Italy; 83 INFN Sezione di Trieste, Università di Trieste, Trieste, Italy; 84 Kyungpook National University, Daegu, Korea; 85 Institute for Universe and Elementary Particles, Chonnam National University, Kwangju, Korea; 86 Hanyang University, Seoul, Korea; 87 Korea University, Seoul, Korea; 88 Department of Physics, Kyung Hee University, Seoul, Republic of Korea; 89 Sejong University, Seoul, Korea; 90 Seoul National University, Seoul, Korea; 91 University of Seoul, Seoul, Korea; 92 Department of Physics, Yonsei University, Seoul, Korea; 93 Sungkyunkwan University, Suwon, Korea; 94 College of Engineering and Technology, American University of the Middle East (AUM), Egaila, Kuwait; 95 Riga Technical University, Riga, Latvia; 96 Vilnius University, Vilnius, Lithuania; 97 National Centre for Particle Physics, Universiti Malaya, Kuala Lumpur, Malaysia; 98 Universidad de Sonora (UNISON), Hermosillo, Mexico; 99 Centro de Investigacion y de Estudios Avanzados del IPN, Mexico City, Mexico; 100 Universidad Iberoamericana, Mexico City, Mexico; 101 Benemerita Universidad Autonoma de Puebla, Puebla, Mexico; 102 University of Montenegro, Podgorica, Montenegro; 103 University of Auckland, Auckland, New Zealand; 104 University of Canterbury, Christchurch, New Zealand; 105 National Centre for Physics, Quaid-I-Azam University, Islamabad, Pakistan; 106 Faculty of Computer Science, Electronics and Telecommunications, AGH University of Science and Technology, Kraków, Poland; 107 National Centre for Nuclear Research, Swierk, Poland; 108 Institute of Experimental Physics, Faculty of Physics, University of Warsaw, Warsaw, Poland; 109 Laboratório de Instrumentação e Física Experimental de Partículas, Lisbon, Portugal; 110 Joint Institute for Nuclear Research, Dubna, Russia; 111 Petersburg Nuclear Physics Institute, Gatchina (St. Petersburg), Russia; 112 Institute for Nuclear Research, Moscow, Russia; 113 Institute for Theoretical and Experimental Physics named by A.I. Alikhanov of NRC ‘Kurchatov Institute’, Moscow, Russia; 114 Moscow Institute of Physics and Technology, Moscow, Russia; 115 National Research Nuclear University ‘Moscow Engineering Physics Institute’ (MEPhI), Moscow, Russia; 116 P.N. Lebedev Physical Institute, Moscow, Russia; 117 Skobeltsyn Institute of Nuclear Physics, Lomonosov Moscow State University, Moscow, Russia; 118 Novosibirsk State University (NSU), Novosibirsk, Russia; 119 Institute for High Energy Physics of National Research Centre ‘Kurchatov Institute’, Protvino, Russia; 120 National Research Tomsk Polytechnic University, Tomsk, Russia; 121 Tomsk State University, Tomsk, Russia; 122 Faculty of Physics and VINCA Institute of Nuclear Sciences, University of Belgrade, Belgrade, Serbia; 123 Centro de Investigaciones Energéticas Medioambientales y Tecnológicas (CIEMAT), Madrid, Spain; 124 Universidad Autónoma de Madrid, Madrid, Spain; 125 Instituto Universitario de Ciencias y Tecnologías Espaciales de Asturias (ICTEA), Universidad de Oviedo, Oviedo, Spain; 126 Instituto de Física de Cantabria (IFCA), CSIC-Universidad de Cantabria, Santander, Spain; 127 University of Colombo, Colombo, Sri Lanka; 128 Department of Physics, University of Ruhuna, Matara, Sri Lanka; 129 CERN, European Organization for Nuclear Research, Geneva, Switzerland; 130 Paul Scherrer Institut, Villigen, Switzerland; 131 ETH Zurich-Institute for Particle Physics and Astrophysics (IPA), Zurich, Switzerland; 132 Universität Zürich, Zurich, Switzerland; 133 National Central University, Chung-Li, Taiwan; 134 National Taiwan University (NTU), Taipei, Taiwan; 135 Department of Physics, Faculty of Science, Chulalongkorn University, Bangkok, Thailand; 136 Physics Department, Science and Art Faculty, Çukurova University, Adana, Turkey; 137 Physics Department, Middle East Technical University, Ankara, Turkey; 138 Bogazici University, Istanbul, Turkey; 139 Istanbul Technical University, Istanbul, Turkey; 140 Istanbul University, Istanbul, Turkey; 141 Institute for Scintillation Materials of National Academy of Science of Ukraine, Kharkov, Ukraine; 142 National Scientific Center, Kharkov Institute of Physics and Technology, Kharkov, Ukraine; 143 University of Bristol, Bristol, UK; 144 Rutherford Appleton Laboratory, Didcot, UK; 145 Imperial College, London, UK; 146 Brunel University, Uxbridge, UK; 147 Baylor University, Waco, USA; 148 Catholic University of America, Washington, DC, USA; 149 The University of Alabama, Tuscaloosa, USA; 150 Boston University, Boston, USA; 151 Brown University, Providence, USA; 152 University of California, Davis, Davis, USA; 153 University of California, Los Angeles, USA; 154 University of California, Riverside, Riverside, USA; 155 University of California, San Diego, La Jolla, USA; 156 Department of Physics, University of California, Santa Barbara, Santa Barbara, USA; 157 California Institute of Technology, Pasadena, USA; 158 Carnegie Mellon University, Pittsburgh, USA; 159 University of Colorado Boulder, Boulder, USA; 160 Cornell University, Ithaca, USA; 161 Fermi National Accelerator Laboratory, Batavia, USA; 162 University of Florida, Gainesville, USA; 163 Florida State University, Tallahassee, USA; 164 Florida Institute of Technology, Melbourne, USA; 165 University of Illinois at Chicago (UIC), Chicago, USA; 166 The University of Iowa, Iowa City, USA; 167 Johns Hopkins University, Baltimore, USA; 168 The University of Kansas, Lawrence, USA; 169 Kansas State University, Manhattan, USA; 170 Lawrence Livermore National Laboratory, Livermore, USA; 171 University of Maryland, College Park, USA; 172 Massachusetts Institute of Technology, Cambridge, USA; 173 University of Minnesota, Minneapolis, USA; 174 University of Mississippi, Oxford, USA; 175 University of Nebraska-Lincoln, Lincoln, USA; 176 State University of New York at Buffalo, Buffalo, USA; 177 Northeastern University, Boston, USA; 178 Northwestern University, Evanston, USA; 179 University of Notre Dame, Notre Dame, USA; 180 The Ohio State University, Columbus, USA; 181 Princeton University, Princeton, USA; 182 University of Puerto Rico, Mayagüez, USA; 183 Purdue University, West Lafayette, USA; 184 Purdue University Northwest, Hammond, USA; 185 Rice University, Houston, USA; 186 University of Rochester, Rochester, USA; 187 Rutgers, The State University of New Jersey, Piscataway, USA; 188 University of Tennessee, Knoxville, USA; 189 Texas A&M University, College Station, USA; 190 Texas Tech University, Lubbock, USA; 191 Vanderbilt University, Nashville, USA; 192 University of Virginia, Charlottesville, USA; 193 Wayne State University, Detroit, USA; 194 University of Wisconsin-Madison, Madison, WI USA; 195 Vienna University of Technology, Vienna, Austria; 196 Institute of Basic and Applied Sciences, Faculty of Engineering, Arab Academy for Science, Technology and Maritime Transport, Alexandria, Egypt; 197 Université Libre de Bruxelles, Brussels, Belgium; 198 Universidade Estadual de Campinas, Campinas, Brazil; 199 Federal University of Rio Grande do Sul, Porto Alegre, Brazil; 200 University of Chinese Academy of Sciences, Beijing, China; 201 Department of Physics, Tsinghua University, Beijing, China; 202 UFMS, Nova Andradina, Brazil; 203 Department of Physics, Nanjing Normal University, Nanjing, China; 204 The University of Iowa, Iowa City, USA; 205 Institute for Theoretical and Experimental Physics named by A.I. Alikhanov of NRC ‘Kurchatov Institute’, Moscow, Russia; 206 Joint Institute for Nuclear Research, Dubna, Russia; 207 Ain Shams University, Cairo, Egypt; 208 Zewail City of Science and Technology, Zewail, Egypt; 209 British University in Egypt, Cairo, Egypt; 210 Purdue University, West Lafayette, USA; 211 Université de Haute Alsace, Mulhouse, France; 212 Erzincan Binali Yildirim University, Erzincan, Turkey; 213 CERN, European Organization for Nuclear Research, Geneva, Switzerland; 214 III. Physikalisches Institut A, RWTH Aachen University, Aachen, Germany; 215 University of Hamburg, Hamburg, Germany; 216 Department of Physics, Isfahan University of Technology, Isfahan, Iran; 217 Brandenburg University of Technology, Cottbus, Germany; 218 Skobeltsyn Institute of Nuclear Physics, Lomonosov Moscow State University, Moscow, Russia; 219 Physics Department, Faculty of Science, Assiut University, Assiut, Egypt; 220 Eszterhazy Karoly University, Karoly Robert Campus, Gyongyos, Hungary; 221 Institute of Physics, University of Debrecen, Debrecen, Hungary; 222 Institute of Nuclear Research ATOMKI, Debrecen, Hungary; 223 MTA-ELTE Lendület CMS Particle and Nuclear Physics Group, Eötvös Loránd University, Budapest, Hungary; 224 Wigner Research Centre for Physics, Budapest, Hungary; 225 IIT Bhubaneswar, Bhubaneswar, India; 226 Institute of Physics, Bhubaneswar, India; 227 G.H.G. Khalsa College, Punjab, India; 228 Shoolini University, Solan, India; 229 University of Hyderabad, Hyderabad, India; 230 University of Visva-Bharati, Santiniketan, India; 231 Indian Institute of Technology (IIT), Mumbai, India; 232 Deutsches Elektronen-Synchrotron, Hamburg, Germany; 233 Sharif University of Technology, Tehran, Iran; 234 Department of Physics, University of Science and Technology of Mazandaran, Behshahr, Iran; 235 INFN Sezione di Bari, Università di Bari, Politecnico di Bari, Bari, Italy; 236 Italian National Agency for New Technologies, Energy and Sustainable Economic Development, Bologna, Italy; 237 Centro Siciliano di Fisica Nucleare e di Struttura Della Materia, Catania, Italy; 238 Università di Napoli ‘Federico II’, Naples, Italy; 239 Riga Technical University, Riga, Latvia; 240 Consejo Nacional de Ciencia y Tecnología, Mexico City, Mexico; 241 IRFU, CEA, Université Paris-Saclay, Gif-sur-Yvette, France; 242 Institute for Nuclear Research, Moscow, Russia; 243 National Research Nuclear University ‘Moscow Engineering Physics Institute’ (MEPhI), Moscow, Russia; 244 St. Petersburg State Polytechnical University, St. Petersburg, Russia; 245 University of Florida, Gainesville, USA; 246 Imperial College, London, UK; 247 P.N. Lebedev Physical Institute, Moscow, Russia; 248 California Institute of Technology, Pasadena, USA; 249 INFN Sezione di Padova, Università di Padova, Università di Trento, Trento, Italy, Padua, Italy; 250 Budker Institute of Nuclear Physics, Novosibirsk, Russia; 251 Faculty of Physics, University of Belgrade, Belgrade, Serbia; 252 Trincomalee Campus, Eastern University, Nilaveli, Sri Lanka; 253 INFN Sezione di Pavia, Università di Pavia, Pavia, Italy; 254 National and Kapodistrian University of Athens, Athens, Greece; 255 Ecole Polytechnique Fédérale Lausanne, Lausanne, Switzerland; 256 Universität Zürich, Zurich, Switzerland; 257 Stefan Meyer Institute for Subatomic Physics, Vienna, Austria; 258 Laboratoire d’Annecy-le-Vieux de Physique des Particules, IN2P3-CNRS, Annecy-le-Vieux, France; 259 Şırnak University, Sirnak, Turkey; 260 Research Center of Experimental Health Science, Near East University, Nicosia, Turkey; 261 Konya Technical University, Konya, Turkey; 262 Faculty of Engineering, Istanbul University-Cerrahpasa, Istanbul, Turkey; 263 Mersin University, Mersin, Turkey; 264 Piri Reis University, Istanbul, Turkey; 265 Adiyaman University, Adiyaman, Turkey; 266 Ozyegin University, Istanbul, Turkey; 267 Izmir Institute of Technology, Izmir, Turkey; 268 Necmettin Erbakan University, Konya, Turkey; 269 Bozok Universitetesi Rektörlügü, Yozgat, Turkey; 270 Marmara University, Istanbul, Turkey; 271 Milli Savunma University, Istanbul, Turkey; 272 Kafkas University, Kars, Turkey; 273 Istanbul Bilgi University, Istanbul, Turkey; 274 Hacettepe University, Ankara, Turkey; 275 Vrije Universiteit Brussel, Brussels, Belgium; 276 School of Physics and Astronomy, University of Southampton, Southampton, UK; 277 IPPP Durham University, Durham, UK; 278 Faculty of Science, Monash University, Clayton, Australia; 279 Bethel University, St. Paul, Minneapolis, USA; 280 Karamanoğlu Mehmetbey University, Karaman, Turkey; 281 Bingol University, Bingol, Turkey; 282 Georgian Technical University, Tbilisi, Georgia; 283 Sinop University, Sinop, Turkey; 284 Mimar Sinan University, Istanbul, Turkey; 285 Erciyes University, Kayseri, Turkey; 286 Texas A&M University at Qatar, Doha, Qatar; 287 Kyungpook National University, Daegu, Korea; 288 CERN, 1211 Geneva 23, Switzerland

## Abstract

The measurement of the luminosity recorded by the CMS detector installed at LHC interaction point 5, using proton–proton collisions at $\sqrt{s}=13\,{\text {TeV}}$ in 2015 and 2016, is reported. The absolute luminosity scale is measured for individual bunch crossings using beam-separation scans (the van der Meer method), with a relative precision of 1.3 and 1.0% in 2015 and 2016, respectively. The dominant sources of uncertainty are related to residual differences between the measured beam positions and the ones provided by the operational settings of the LHC magnets, the factorizability of the proton bunch spatial density functions in the coordinates transverse to the beam direction, and the modeling of the effect of electromagnetic interactions among protons in the colliding bunches. When applying the van der Meer calibration to the entire run periods, the integrated luminosities when CMS was fully operational are 2.27 and 36.3 $\,\text {fb}^{-1}$ in 2015 and 2016, with a relative precision of 1.6 and 1.2%, respectively. These are among the most precise luminosity measurements at bunched-beam hadron colliders.

## Introduction

Luminosity, $\mathcal {L}$, is a key parameter at particle colliders. Along with the energy available in the center-of-mass system, it is one of the two main figures of merit that quantify the potential for delivering large data samples and producing novel massive particles. The instantaneous luminosity $\mathcal {L} (t)$ is the process-independent ratio of the rate *R*(*t*) of events produced per unit of time ${\text {d}}t$ to the cross section $\sigma$ for a given process. The fundamental limitations on precise predictions for these cross sections (e.g., from quantum chromodynamics) motivate the techniques used for luminosity measurements at various types of colliders. The precise determination of the integrated luminosity, $\int \mathcal {L} (t){\text {d}}t$, has proven particularly challenging at hadron colliders, with an achieved precision typically ranging from 1 to 15% [1]. The “precision frontier” target of 1% [2] does not reflect a fundamental limitation, but rather results from a variety of uncorrelated sources of systematic uncertainty with typical magnitudes of 0.1–0.5%. In this paper, we report the precise determination of the absolute luminosity at the CERN LHC interaction point (IP) 5 with the CMS detector [3], using data from proton–proton (${\text {p}}{\text {p}}$ ) collisions at $\sqrt{s}=13\,{\text {TeV}}$ collected in 2015 and 2016.

A central component of the physics program at the LHC consists of measurements that can precisely test the validity of standard model (SM) predictions, e.g., cross sections for the production of electroweak gauge bosons [4, 5] or top quark pairs [6, 7]. A good understanding of the luminosity is critical to minimize the systematic uncertainty in these measurements. The uncertainty in the luminosity measurement is often the dominant systematic uncertainty [5–7], motivating an effort to improve its precision.

Stable luminosity information is also crucial to the efforts of the LHC operators to optimize the performance of the accelerator [8, 9]. In this context, it is important to provide luminosity information in real time at a high enough frequency to facilitate rapid optimization. The ability to measure the luminosity of individual bunch crossings (bunch-by-bunch luminosity) is also necessary so that the distribution of number of collisions per crossings is known to the experiments. This information is important when preparing simulations as well as optimization of thresholds to keep event-recording rates near data acquisition design targets.

An absolute luminosity scale is obtained with good accuracy using the direct method of van der Meer (vdM) scans [10–13]. In these scans, the transverse separation of the two beams is varied over time and the resulting rate of some physical observables (e.g., number of charged particles passing through a silicon detector or energy deposited in a calorimeter) as a function of separation is used to extract the effective beam size. The absolute luminosity at one point in time can then be calculated from measurable beam parameters – namely, the transverse spatial widths of the overlap of the beams and the number of protons in each beam. To achieve the desired accuracy in the absolute luminosity calibration, the vdM scans are typically performed under carefully tailored conditions and with beam parameters optimized for that purpose [1], in conjunction with processing the input from accelerator instrumentation and multiple detector systems. A relative normalization method is then needed to transfer the absolute luminosity calibration to the complete data-taking period. To this end, for a given subdetector, the cross section $\sigma _{\mathrm {vis}}$ in the “visible” phase space region, defined by its acceptance, is measured for several observables. The integrated luminosity is obtained from the $\sigma _{\mathrm {vis}}$-calibrated counts accumulated for a given period of data taking. Changes in the detector response over time can result in variations in $\sigma _{\mathrm {vis}}$, which could appear as nonlinearity and/or long-term instability in the measured luminosity.

To address these challenges, CMS employs a multifaceted approach, in which measurements from various individual subsystems are used to produce a final luminosity value with high precision, good linearity, and stability. Several methods and independent detectors are used to provide redundancy and to minimize any bias originating from detector effects.

The LHC orbit is divided into a total of 3564 time windows 25$\text { ns}$ long (bunch crossing slots), each of which can potentially contain a colliding bunch. However, the total number of filled bunch crossings is limited by design to a maximum of 2808 by the choice of the beam production scheme in the injectors and constraints from the rise times of injection and extraction kicker magnets in the various accelerators involved [14]. Furthermore, the length of the injections in 2015 and 2016 was limited by the maximal tolerable heat load in the arcs due to electron clouds (2015) and safety considerations in the LHC injection system with very luminous beams (2016) [15]. The bunch crossings are numbered with an identification number (BCID) in the range 1–3564. The specific pattern of filled and empty bunch crossings used in a single fill is known as the “filling scheme”; a typical filling scheme is composed of long strings of consecutive bunches, up to 72 bunches long, called a “train”, with the individual trains separated by gaps of varying lengths. Generally, filling schemes also include some number of noncolliding bunch crossings, where one beam is filled but the other remains empty; these can be used to study effects from beam-induced background. The two LHC beams are designated “beam 1” and “beam 2”, where beam 1 (beam 2) circulates in the clockwise (counterclockwise) direction, as viewed from above [14].

For Run 2 of the LHC, the period from 2015 to 2018 featuring ${\text {p}}{\text {p}}$ collisions at $\sqrt{s}=13\,{\text {TeV}}$, the CMS luminosity systems were significantly upgraded and expanded. We report the results for the first two years [16], in which the operational conditions feature a wide range in the number of colliding bunches $n_{\mathrm {b}}$ and instantaneous luminosity, reaching a maximum of 2232 and 2208, and $0.5\times \text {10}^\text {34}$ and $1.5\times \text {10}^\text {34}\,\text {cm}^{-2}\,\text {s}^{-1}$ in 2015 and 2016, respectively. In the majority of ${\text {p}}{\text {p}}$ LHC fills in Run 2, the bunches are spaced 25$\text { ns}$ apart. The initial Run 2 data set delivered with a bunch spacing of 50$\text { ns}$ is negligibly small [17], and hence not included in this paper. In this paper, “pileup” refers to the total number of ${\text {p}}{\text {p}}$ interactions in a single bunch crossing, and “out-of-time pileup” refers to additional ${\text {p}}{\text {p}}$ collisions in nearby bunches. For a total inelastic ${\text {p}}{\text {p}}$ cross section of 80$\text { mb}$  [18, 19], the pileup during nominal physics data-taking conditions in 2015 (2016) extended from 5 to 35 (10 to 50) with an expected average ($\mu$) of about 14 (27) ${\text {p}}{\text {p}}$ interactions.

This paper is structured as follows. In Sect. 2 the CMS detector is described with special emphasis on the subdetectors used to derive observables for luminosity estimation, and in Sect. 3 we review the methods to obtain the luminosity information. Section 4 describes the vdM scan calibration method and the associated systematic uncertainty. Sections 5 and 6 outline the corrections applied to the luminosity algorithms and their resulting performance, respectively. Finally, Sect. 7 outlines the sources of corrections and the associated systematic uncertainties, and presents the main results. A summary is given in Sect. 8.

## The CMS detector

The CMS detector is a multipurpose apparatus designed to study high-$p_{\mathrm {T}}$ physics processes in ${\text {p}}{\text {p}}$ collisions, as well as a broad range of phenomena in heavy ion collisions. The central element of CMS is a 3.8$\text { T}$ superconducting solenoid, 13$\text { m}$ in length and 6$\text { m}$ in diameter. Within the solenoid volume are – in order of increasing radius from the beam pipe – a silicon pixel and strip tracker of high granularity for measuring charged particles up to pseudorapidity ($\eta$) of $\pm 2.5$; a lead tungstate crystal electromagnetic calorimeter for measurements of the energy of photons, electrons, and the electromagnetic component of hadronic showers (“jets”); and a brass and scintillator hadron calorimeter, each composed of a barrel and two endcap sections, for jet energy measurements. The forward hadron (HF) calorimeter uses steel as an absorber and quartz fibers as the sensitive material. The two halves of the HF are located 11.2$\text { m}$ from the interaction region, one on each end, and together they provide coverage in the range $3.0< |\eta | < 5.2$, hence extending the pseudorapidity coverage provided by the barrel and endcap detectors. Outside the magnet, and within the range $|\eta | < 2.4$, is the muon system [20], which is embedded in the iron flux-return yoke. It is composed of detection planes made using three technologies: drift tubes (DTs) in the barrel, cathode strip chambers (CSCs) in the endcaps, and resistive plate chambers (RPCs) both in the barrel and in the endcaps.

Events of interest for physics are selected using a two-tiered trigger system [21]. The first-level trigger, composed of custom hardware processors, uses information from the calorimeters and muon detectors to select events at a rate of around 100$\text { kHz}$. The second level, known as the high-level trigger, consists of a farm of processors running a version of the full event reconstruction software optimized for fast processing, and reduces the event rate to around 1$\text { kHz}$ before data storage.

Several subdetectors, although not part of the main CMS data acquisition (DAQ) system, provide additional inputs (e.g., binary logic signals) to the triggering system. The two beam monitors closest to the IP for each LHC experiment, the Beam Pick-up Timing for eXperiments (BPTX) detectors [22], are reserved for timing measurements. They are located on either side of IP 5 at a distance of approximately 175$\text { m}$. The BPTX system can be used to provide a set of zero-bias events (i.e., events from nominally colliding bunch crossings but without a requirement for any specific activity in the event) by requiring a coincidence between the two BPTX sides. To suppress noise in triggers with high background, the presence of this coincidence is typically required [21].

The knowledge of the integrated luminosity requires stability over long periods of time, and hence benefits greatly from redundant measurements whose combination can lead to an improved precision. To that end, several upgrades were completed during the first LHC long shutdown (LS1), the transition period between LHC Run 1 (2009–2012) and Run 2. The main luminosity subdetectors (luminometers) in Run 1 were the silicon pixel detector and the HF. The HF back-end electronics, which were upgraded during LS1, consist of two independent readout systems: a primary readout over optical links for physics data taking, and a secondary readout using Ethernet links, explicitly reserved for luminosity data. In addition, two other luminometers were designed, constructed, and commissioned: the Pixel Luminosity Telescope (PLT) [23] and the Fast Beam Conditions Monitor (BCM1F) [24]. Finally, a separate DAQ system was developed that is independent of the central DAQ system [21, 25], so that HF, PLT, and BCM1F data, as well as LHC beam-related data, are collected and stored in a time- rather than event-based manner.

**Fig. 1:**
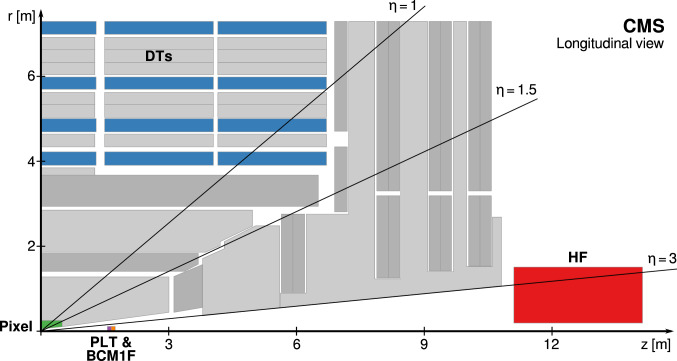
Schematic cross section through the CMS detector in the *r*-*z* plane. The main luminometers in Run 2, as described in the text, are highlighted, showing the silicon pixel detector, PLT, BCM1F, DTs, and HF. The two RAMSES monitors used as a luminometer in Run 2 are located directly behind HF. In this view, the detector is symmetric about the horizontal and vertical axes, so only one quarter is shown here. The center of the detector, corresponding to the approximate position of the ${\text {p}}{\text {p}}$ collision point, is located at the origin. Solid lines represent distinct $\eta$ values

The luminometers, along with the accompanying algorithms used to estimate the instantaneous luminosity in Run 2, are briefly described in the following. Figure 1 shows an overview of the position of these luminometers within CMS. A more detailed description of the rest of the CMS detector, together with a definition of the coordinate system used and the relevant kinematic variables, is reported in Ref. [3].

### Silicon pixel cluster counting

The pixel cluster counting (PCC) method, which uses the mean number of pixel clusters in the silicon pixel detector, exploits the very large number of pixels in the inner part of the CMS tracking system. The number of pixels in 2015–2016 was about $7\times \text {10}^\text {7}$, which means that the probability of a given pixel being hit by two different charged particles from the same bunch crossing is exceedingly small. The mean number of pixel clusters in simulated zero-bias events is of the order of 100 per ${\text {p}}{\text {p}}$ collision, although the precise mean depends on the fraction of the detector used for a given data set. Assuming each pixel cluster comprises five pixels and using a typical pileup for the 2016 running of $\mu = 27$, the fraction of pixels hit in a typical bunch crossing is roughly:

1 $$\begin{aligned} f = \frac{N^{\text {hit}}_{\mathrm {pixel}} }{ N^{\text {total}}_{\mathrm {pixel}}} \simeq \frac{ 100 \times 5 \times 27}{ 7 \times 10^7} = 0.02\%. \end{aligned}$$

**Fig. 2:**
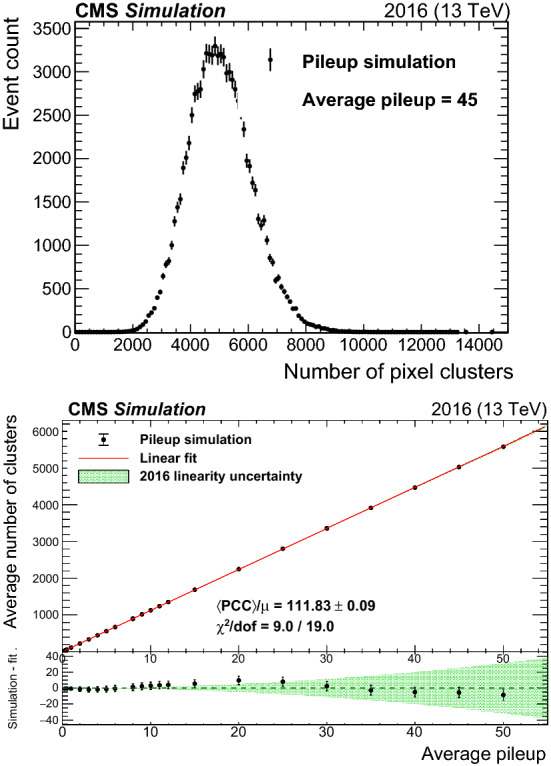
The upper plot shows the number of pixel clusters and their statistical uncertainty from simulation of pileup following a Poisson distribution with a mean of 45. The lower plot shows the mean number of pixel clusters from simulation as a function of mean pileup. The red curve is a first-order polynomial fit with slope and $\chi ^2/\text {dof}$ values shown in the legend. Only pixel modules considered for the PCC measurement in data are included. The lower panel of the lower plot shows the difference between the simulation and the linear fit in black points. The green band is the final linearity uncertainty for the 2016 data set

The probability of accidental overlap between pixel clusters is correspondingly small, and, as a consequence, the number of pixel clusters per bunch crossing is linearly dependent on pileup, and therefore an accurate measure of instantaneous luminosity. Simulated ${\text {p}}{\text {p}}$ collision events that contain only in-time pileup and detector noise are generated using pythia version 8.223 [19] with the CUETP8M1 [18, 26] tune. The simulated events include a full simulation of the CMS detector response based on Geant4  [27]. For the sake of simplicity, the number of pileup interactions present in each simulated event is randomly generated from a Poisson distribution with $\mu$ up to 50. Figure 2 shows a representative PCC distribution at $\mu =45$ and the average PCC as a function of $\mu$. The latter distribution is fitted with a first-order polynomial, assuming no correlations among different values of $\mu$. Good agreement is seen based on the estimated goodness-of-fit $\chi ^2$ per degree of freedom (dof) value of about 0.5 [28], indicating linearity under simulated conditions.

Only the components (modules) of the pixel subdetector that are stable for the entire period of data taking are used for the PCC rate measurements, excluding pixel modules known to be defective or significantly affected by the limited size of the readout buffer [29]. The measured $\sigma _{\mathrm {vis}}$ for PCC, $\sigma _{\mathrm {vis}}^{\text {PCC}}$, therefore depends on the data-taking period (i.e., one calibration per year).

### Primary vertex counting

The primary vertex counting (PVC) method uses the vertices that have been reconstructed using the tracks in the CMS detector. For this method, a good primary vertex is defined to be one with 11 or more tracks. This requirement is sufficient to suppress spurious vertices [29], and results in better vertex resolution.

The PVC method is simple and robust, but suffers from mild nonlinearity effects when there are many collisions in a single bunch crossing. There are two competing effects. In one effect, primary vertices from two collisions occurring close to one another in space are merged, leading to an undercounting of vertices. In the other effect, the very large numbers of tracks associated with numerous collisions can produce spurious vertices, leading to overcounting. The precision with which these effects are understood falls short of the $\approx 1\%$ level needed for luminosity studies. However, during vdM scans these effects are minimal because of the very low pileup, and so PVC is very useful as a validation tool for the vdM analysis in the measurement of beam-dependent parameters.

### Forward hadron calorimeter

The HF luminosity measurement uses the separate readout described above, so the measurement can be performed at the full 40$\text { MHz}$ bunch crossing rate. The back-end electronics upgrade during LS1 added new electronics using field-programmable gate array (FPGA) technology such that several features of the readout were separately programmable for luminosity histogramming, i.e., identifying and counting the readout channels. Although the whole HF is capable of being read out for luminosity use, only the two outer rings in $\eta$ are used to ensure uniform occupancy and minimize minor nonlinearities expected from simulation.

The computation of the HF observable is based on the occupancy method (HFOC). In this method, the fraction of channel measurements above an analog-to-digital converter (ADC) threshold is used for each bunch slot in a configurable time window. The ADC threshold is set high enough to avoid most noise and as low as possible otherwise. Both the ADC threshold and the integration time of the histograms between readouts are configurable, but they were fixed during data taking in 2015 and 2016. The number of valid measurements is also stored, so the fraction of events with hits above threshold can be computed.

### Pixel Luminosity Telescope

The PLT is a dedicated system for measuring luminosity using silicon pixel sensors, installed in Run 2, at the beginning of 2015. There are a total of 48 sensors arranged into 16 “telescopes”, eight at either end of CMS outside the pixel endcap. Each telescope contains three sensor planes which are arranged in a triplet that faces the IP. The sensors measure $8{\times }8 \text { mm} ^2$, divided into 80 rows and 52 columns, although only the central region of the sensors is used to reduce the contribution from background. The PLT measures the rate of triple coincidences, where a hit is observed in all three planes, typically corresponding to a track from a particle originating at the IP. The overall mean rate for PLT is estimated using the fraction of events where no triple coincidences are observed (as described in Sect. 3) in order to avoid potential systematic effects from overlapping tracks being counted as a single hit.

### Fast beam conditions monitor

The BCM1F measures luminosity and beam-induced background separately. It consists of a total of 24 sensors mounted on the same carriage as the PLT. Single-crystal diamond sensors are used with split-pad metallization. Each sensor has two readout channels to keep the overall occupancy low, given the experimental conditions in Run 2. The BCM1F features a fast readout with 6.25$\text { ns}$ time resolution. The precise time measurement allows hits from collision products to be separated from beam-induced background hits, while the incoming background is separated in time from the outgoing collision products due to the position of BCM1F 1.8 m from the center of CMS.

### Drift tube muon detector

The luminosity measurement based on the DT muon detector [20] is based on an efficient trigger on a low-background physics object: muons produced in the CMS barrel. Muon track segments from barrel muon DT stations are sent every bunch crossing to track finder hardware, where tracks are built and later used to generate first level triggers. The number of tracks in time windows of approximately 23 s is read out and stored in a database. These data are used to estimate luminosity. The rate of muons in the DTs is significantly lower than the rate for most other observables from other luminometers. Thus, there are not enough muon tracks during the vdM scans to provide a precise measurement of $\sigma _{\mathrm {vis}}$, and so the system must be calibrated to the normalized PCC luminosity measurement. On the other hand, the muon candidate rate has been observed to be linear with luminosity and rather stable over time. The luminosity data of this system are integrated over all bunches.

### Radiation monitoring system for the environment and safety

The Radiation Monitoring System for the Environment and Safety (RAMSES) is a monitoring subsystem of the unified supervisory CERN system [30, 31]. There are 10 ionization chambers filled with air at atmospheric pressure that are used as monitors installed in the CMS experimental cavern. They are sensitive to ionizing radiation and can monitor the ambient dose equivalent rate. Thus, they generate alarms and interlocks to ensure the safety of the personnel. This system is maintained and calibrated by the LHC radiation protection group.

While not designed as a luminometer, the two chambers with the highest rates (designated PMIL55X14 and PMIL55X15) have been used to produce a luminosity measurement with good linearity and stability over time. However, similarly to the DT luminosity measurement, the overall rates are too low for bunch-by-bunch measurements or extracting an absolute calibration during vdM scans. The RAMSES luminosity is thus calibrated to the normalized PCC luminosity measurement and is used as an additional measurement for assessing the luminometer stability with time.

## Luminosity determination algorithms

Each bunch crossing gives rise to a certain number of ${\text {p}}{\text {p}}$ interactions. In a given luminometer, each interaction results in some number of observables (e.g., hits, tracks, or clusters). If one averages over several unbiased measurements, the mean number of observables is

2 $$\begin{aligned} \begin{aligned} \langle N_{\mathrm {observables}} \rangle&= \langle N_{\mathrm {observables/interaction}} \rangle \langle N_{\mathrm {interactions}} \rangle \\&\equiv \langle N_{\mathrm {observables/interaction}} \rangle \mu , \end{aligned} \end{aligned}$$

where the average number of interactions per bunch crossing is denoted by $\mu$, in keeping with the Poisson nature of the underlying probability distribution. Typically these observables are averaged over seconds or tens of seconds.

To measure the instantaneous luminosity, we use the fact that $\mu$ is proportional to the single-bunch crossing instantaneous luminosity $\mathcal {L} _{\mathrm {b}}$ via:

3 $$\begin{aligned} \mu = \frac{\sigma \mathcal {L} _{\mathrm {b}}}{\nu _r}, \end{aligned}$$

where $\nu _r =11\,245.6\text { Hz}$ is the LHC revolution frequency during collisions, and $\sigma$ is the total interaction cross section. At the LHC, $\mathcal {L} _{\mathrm {b}}$ is typically expressed in units of $\text {Hz}/\upmu \text {b} \equiv 10^{30}\,\text {cm}^{-2}\,\text {s}^{-1}$.

Two algorithms have been developed for extracting the instantaneous luminosity. One method is rate-scaling, where the raw rate of observables is scaled with calibration constants to the luminosity. Rearranging Eqs. (2) and (3), one can estimate the instantaneous luminosity using the average number of observables at a given time:

4 $$\begin{aligned} \mathcal {L} _{\mathrm {b}} = \frac{\langle N_{\mathrm {observables}} \rangle }{\langle N_{\mathrm {observables/interaction}} \rangle } \frac{\nu _r}{\sigma } \equiv \langle N_{\mathrm {observables}} \rangle \frac{\nu _r}{\sigma _{\mathrm {vis}}}. \end{aligned}$$

Luminosity is estimated from PCC, PVC, DT, and RAMSES data using the rate-scaling algorithm.

The second method (zero counting) uses the average fraction of bunch crossings where no observables in a detector are produced. This zero fraction is then used to infer the mean number of observables per bunch crossing. The principal advantage of the zero-counting method is that it is not affected by cases where two or more separate signals overlap in the detector and produce only one reconstructed observable.

Assuming that the probability of no observables in a single collision is *p*, then the probability of no observables seen in a bunch crossing with *k* interactions is thus simply $p^k$. Averaged over a large number of bunch crossings, with the number of interactions per bunch crossing distributed according to a Poisson distribution of mean $\mu$, the expected fraction of events with zero observables recorded, $\langle f_0 \rangle$, can be expressed as:

5 $$\begin{aligned} \langle f_0 \rangle = \sum _{k=0}^{\infty }\frac{\mathrm {e}^{-\mu }\mu ^k}{k!}p^k = \mathrm {e}^{-\mu \left( 1-p\right) }. \end{aligned}$$

The logarithm of Eq. (5) is proportional to the mean number of ${\text {p}}{\text {p}}$ interactions per bunch crossing, and hence to the $\mathcal {L} _{\mathrm {b}}$ according to Eq. (3):

6 $$\begin{aligned} \mathcal {L} _{\mathrm {b}} = \mu \frac{\nu _r}{\sigma } = -\ln {\langle f_0 \rangle }\frac{1}{1-p} \frac{\nu _r}{\sigma } \equiv -\ln {\langle f_0 \rangle } \frac{\nu _r}{\sigma _{\mathrm {vis}}}. \end{aligned}$$

The actual value of *p* does not need to be known beforehand, since it is effectively absorbed in $\sigma _{\mathrm {vis}}$, although it could be extracted from the measured $\sigma _{\mathrm {vis}}$ value. The raw inputs from HFOC, PLT, and BCM1F are converted to luminosity using the zero-counting method.

## Absolute luminosity calibration

Any luminometer requires an externally determined absolute calibration. Approximate $\sigma _{\mathrm {vis}}$ values can be obtained using Monte Carlo (MC) simulation, but these ultimately rely on theory, i.e., the inelastic ${\text {p}}{\text {p}}$ cross section, and are not expected to be reliable at the percent level that represents the target accuracy for the CMS luminosity measurement. At the LHC, the precision of theoretical predictions for SM processes is typically limited by the knowledge of the parton distribution functions in the proton. Although methods independent of theoretical assumptions have been proposed at the expense of introducing correlations between low- and high-$\mu$ data-taking periods [32], a more precise and purely experimental method to determine the luminosity is based on the vdM scan technique, which is used in this paper.

Beam-separation scans are therefore performed to obtain calibrated $\sigma _{\mathrm {vis}}$ for the luminosity measurement. These were pioneered by Simon van der Meer at the ISR [10], extended by Carlo Rubbia to the case of a collider with bunched beams [11], and have been extensively used by all four major LHC experiments [12, 13]. The key principle of the vdM scan method is to infer the beam-overlap integral from the rates measured at different beam separations – provided the beam displacements are calibrated as absolute distances – as opposed to measuring the bunch density functions directly. The basic formalism is described in the following.

### The van der Meer method

The instantaneous luminosity for a single colliding bunch pair in a colliding-beam accelerator is given by:

7 $$\begin{aligned} \mathcal {L} _{\mathrm {b}} = \frac{\nu _r n_1 n_2 }{A_{\mathrm {eff}}}, \end{aligned}$$

where $n_1$ and $n_2$ are the numbers of particles in each of the two bunches, and $A_{\mathrm {eff}}$ is the effective area of overlap between the bunches. In general, each of the bunches will be distributed in the plane transverse to the beam direction, in which case $1/A_{\mathrm {eff}}$ can be replaced by an overlap integral of the bunch densities, i.e.,

8 $$\begin{aligned} \mathcal {L} _{\mathrm {b}} = \nu _r n_1 n_2 \iint \rho _1(x,y) \rho _2(x,y) {\text {d}}x {\text {d}}y, \end{aligned}$$

where *x* and *y* represent the horizontal and vertical coordinates in the plane transverse to the beams, and $\rho _1$ and $\rho _2$ are the normalized two-dimensional density distributions for the two bunches. Here, we have integrated over time and the longitudinal coordinate *z*.

If one assumes that the bunch profiles can be factorized into terms depending only on *x* and *y* [10, 11], then $\rho _i$ can be written as the product of one-dimensional density functions of the form $\rho _i(x,y) = f_i(x) g_i(y)$ ($i=1,2$), and $1/A_{\mathrm {eff}}$ can be written

9 $$\begin{aligned} \frac{1}{A_{\mathrm {eff}}} = \int f_1(x) f_2(x) {\text {d}}x \int g_1(y) g_2(y) {\text {d}}y \equiv \frac{1}{W_{\mathrm {eff}}} \frac{1}{H_{\mathrm {eff}}}, \end{aligned}$$

where $W_{\mathrm {eff}}$ and $H_{\mathrm {eff}}$ are the effective width and the effective height of the luminous region. For the ideal case of Gaussian-distributed bunches with the same width in both beams and undergoing head-on collisions, Eq. (8) reduces to:

10 $$\begin{aligned} \mathcal {L} _{\mathrm {b}} = \frac{\nu _r n_1 n_2 }{ 4 \pi \sigma _x \sigma _y}, \end{aligned}$$

where $\sigma _x$ and $\sigma _y$ are the root-mean-square (RMS) widths of the horizontal and vertical bunch profiles in either beam, respectively. In the case of round beams, $\sigma _x=\sigma _y \equiv \sigma _{\mathrm {b}} \equiv \sqrt{\smash [b]{\epsilon _{\mathrm {N}} \beta ^{*}/\gamma }}$, where $\epsilon _{\mathrm {N}}$ is the so-called normalized emittance, $\gamma$ the relativistic Lorentz factor, and $\beta ^{*}$ corresponds to the value of the optical function $\beta$ at the IP [33].

We designate the luminosity when the beams are displaced with respect to each other by an amount *w* in the *x* direction, or an amount *h* in the *y* direction, as $\mathcal {L} (w,h)$. As shown in Ref. [10], when a separation scan is performed in the *x* direction, in which *w* is varied in a systematic way from $-\infty$ to $+\infty$, the effective width can be determined from:

11 $$\begin{aligned} W_{\mathrm {eff}} = \frac{\iint f_1(x) f_2(x-w) {\text {d}}x {\text {d}}w }{ \int f_1(x) f_2(x) {\text {d}}x } = \frac{\int \mathcal {L} _{\mathrm {b}} (w,0) {\text {d}}w }{ \mathcal {L} _{\mathrm {b}} (0,0)}, \end{aligned}$$

where common normalization factors have been canceled in the second step. Similarly, if a scan is performed in the *y* direction, the effective beam-overlap height is given by

12 $$\begin{aligned} H_{\mathrm {eff}} = \frac{\iint g_1(y) g_2(y-h) {\text {d}}y {\text {d}}h }{ \int g_1(y) g_2(y) {\text {d}}y } = \frac{\int \mathcal {L} _{\mathrm {b}} (0,h) {\text {d}}h }{ \mathcal {L} _{\mathrm {b}} (0,0)}. \end{aligned}$$

For Gaussian-distributed bunches, the resulting scan curves, $\mathcal {L} (w,0)$ and $\mathcal {L} (0,h)$, are also Gaussian with RMS widths of $\varSigma _x= W_{\mathrm {eff}} = \sqrt{2}\sigma _x$ and $\varSigma _y = H_{\mathrm {eff}} = \sqrt{2}\sigma _y$, yielding

13 $$\begin{aligned} \mathcal {L} _{\mathrm {b}} = \frac{\nu _r n_1 n_2 }{ 2 \pi \varSigma _x \varSigma _y}. \end{aligned}$$

Equations (11) and (12) are quite general, and do not depend on the assumption of Gaussian-distributed bunches. Indeed, it is frequently the case that simple Gaussians do not provide an adequate description of the scan-curve data. In such cases, we use double-Gaussian functions of the form

14 $$\begin{aligned} f(x) = \frac{1}{\sqrt{2\pi }}\Bigg [\frac{\epsilon _{x}}{\sigma _{1x}}\exp {\Bigg (-\frac{x^2}{2\sigma _{1x}^2}\Bigg )} + \frac{1-\epsilon _{x}}{\sigma _{2x}}\exp {\Bigg (-\frac{x^2}{2\sigma _{2x}^2}\Bigg )}\Bigg ],\nonumber \\ \end{aligned}$$

where $\epsilon _{x}$ is the fraction of the Gaussian with width $\sigma _{1x}$. Normally the Gaussian with the smaller width $\sigma _{1x}$ is considered the core Gaussian, while the Gaussian with the larger width $\sigma _{2x}$ is used to fit the tails of the scan curve. Similar relations apply for the *y* coordinate. The effective value of $\varSigma _i$ ($i=x,y$) is then given by

15 $$\begin{aligned} \varSigma _i = \frac{\sigma _{1i}\,\sigma _{2i}}{\epsilon _i\sigma _{2i}+ (1-\epsilon _i)\sigma _{1i}}. \end{aligned}$$

To calibrate a given luminosity algorithm, the absolute luminosity computed from beam parameters via Eq. (13) is used in conjunction with Eq. (3) to obtain

16 $$\begin{aligned} \sigma _{\mathrm {vis}} = \mu _{\mathrm {vis}} \frac{2 \pi \varSigma _x \varSigma _y }{ n_1 n_2}, \end{aligned}$$

where $\mu _{\mathrm {vis}}$ is the visible interaction rate. In this analysis, $\mu _{\mathrm {vis}}$ is taken as the arithmetic mean of the peak values from $\mathcal {L} (w,0)$ and $\mathcal {L} (0,h)$ in scans that are performed sufficiently close in time to minimize the impact of varying bunch distributions over the course of a fill. Equation (16) therefore provides a direct calibration of the visible cross section for each algorithm in terms of $\varSigma _x \varSigma _y$ and $n_1 n_2$.

In the LHC, bunches typically cross at a small angle $\phi$ in the horizontal plane at IP 5. This introduces a reduction in the luminosity relative to the case of head-on collisions [1], given by:

17 $$\begin{aligned} \frac{\mathcal {L}}{\mathcal {L} _0} = \left[ 1 + \left( \frac{\sigma _z}{\sigma _x} \tan \frac{\phi }{2} \right) ^2 \right] ^{-1/2}, \end{aligned}$$

where $\sigma _x$ is the width of the luminous region in the crossing plane and $\sigma _z$ is the width in the longitudinal direction. For typical LHC physics running conditions in 2016, $\phi \simeq 140~\upmu$rad, $\sigma _x \simeq 12\,\upmu \text {m}$, and $\sigma _z \simeq 8\text { cm}$, and so the reduction from Eq. (17) is around 10% [34]. The vdM scans are typically carried out under special conditions, where $\phi =0$, as described in the following. The values of $\sigma _{\mathrm {vis}}$ do not depend on the crossing angle.

### Analysis of vdM scan data

While $\sigma _{\mathrm {vis}}$ does not depend on beam conditions, the LHC delivers beams under special conditions to improve the precision of measurements and to reduce systematic effects. The vdM filling schemes are characterized by a low number of colliding bunch pairs at IP 5 ($n_{\mathrm {b}} =30$–50). The bunches are widely separated from each other in the LHC orbit, to reduce the effect of afterglow (as discussed in Sect. 5.1). Special beam optics with $\beta ^{*} \approx 19\text { m}$ and transverse emittance of $\epsilon _{\mathrm {N}} \approx 3.0\,\upmu \text {m}$ are implemented to produce a relatively large bunch size of approximately $\sigma _{\mathrm {b}} = 100\,\upmu \text {m}$. Large bunches reduce the impact of vertex reconstruction resolution in analyses where vertex positions are utilized. A crossing angle of 0 is used for collisions at IP 5 in vdM scans. To minimize the effect of potential nonlinear response in the luminometers, the target pileup is set to $\mu \approx 0.6$, which is 1–2 orders of magnitude lower than typical physics fills. To achieve that goal, in addition to the large beam size, the beams have relatively low intensities, which typically begin at (8–9)$\times \text {10}^\text {10}$ protons per filled bunch, resulting in a total intensity of (3.5–4.0)$\times \text {10}^\text {12}$ per beam for 44 bunches.

The total beam intensities are measured with the DC current transformers (DCCT) [35], and the bunch currents measured with the fast beam current transformers (FBCT) [36], and cross-checked with the longitudinal density monitors (LDMs) [37, 38] and the beam quality monitors [39]. Because of the low beam intensity and low collision rate, the luminosity remains nearly constant over the course of time, in contrast to typical physics fills [9]. The beam orbit is monitored using two systems, the Diode Orbit and Oscillation (DOROS) beam position monitors (BPMs) [40] located near IP 5, and the BPMs located in the LHC arcs adjacent to CMS (referred to as “LHC arc BPMs”). The latter are transformed to a beam position at IP 5 using the LHC optics files that are centrally provided by LHC operators [41]. The orbit is also tracked using the movements of the luminous region at IP 5 based on the vertices reconstructed with the CMS tracker.

**Fig. 3:**
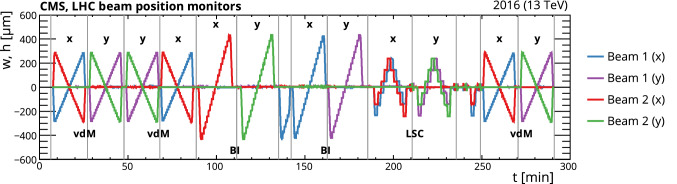
Relative change in the positions of beams 1 and 2 measured by the DOROS BPMs during fill 4954 in the horizontal (*x*) or vertical (*y*) directions, as a function of the time elapsed from the beginning of the program. The gray vertical lines delineate vdM, BI, or LSC scans

The vdM scan program at IP 5 consists of a series of *x*-*y* scan pairs. Figure 3 shows the progression of these scans in a calibration fill, with the beam displacement measured by the DOROS BPMs [40, 42].

Typical scan sessions consist of at least three vdM scan pairs, with one scan in each of the transverse coordinates per pair. There are two at the start of the fill and another at the end of fill. In the absence of systematic effects, all scans are expected to produce compatible results. In each pair, the scans are typically performed first in the *x* and then in the *y* direction, although sometimes the pair is performed in the opposite order. In the vdM scans, the two beams are separated by up to 6$\sigma _{\mathrm {b}}$, and scanned across one another in a sequence of 25 steps of 30$\text { s}$ each to obtain a statistically significant measurement.

Dedicated length scale calibration (LSC) scans (described in Sect. 4.3.4), which are used to calibrate the distance by which the steering magnets displace the beams, are also performed typically close in time to the rest of the scans and using the same collision optics configuration. The LSC implemented at IP 5 is of a constant-separation type, in which the two beams are positioned at $-2.5$ and $-1.5\sigma _{\mathrm {b}}$ relative to nominal and moved together forward in steps of 1$\sigma _{\mathrm {b}}$, maintaining the 1$\sigma _{\mathrm {b}}$ separation between the two beams, until they reach the $+2.5\sigma _{\mathrm {b}}$ point. Then, their positions are swapped, and they are moved together backward in $-1 \sigma _{\mathrm {b}}$ steps back to $-2.5\sigma _{\mathrm {b}}$. The scan is performed once in the *x* direction and once in the *y* direction, with a total of 10 steps of 60$\text { s}$ in each direction. The LSC scans are performed with successive forward and backward displacements for multiple measurements under slightly different conditions in case there are compounding effects that limit precision. The transverse position of the luminous region is needed for this calibration and is measured using reconstructed primary vertices in CMS data.

To test the assumption of transversely factorizable bunch profiles in Eq. (9), four dedicated beam-imaging (BI) scans are performed, one for each beam and each transverse direction. One beam is kept fixed at its head-on position, while the other is moved and scanned in 19 steps from $-4.5$ to $+4.5\sigma _{\mathrm {b}}$ along *x* or *y* with a duration of 40$\text { s}$ per step. Primary vertices are reconstructed, and their positions are then analyzed to perform a global fit to derive the transverse bunch density distributions of the beams (as discussed in Sect. 4.4.1). The BI scans are also analyzed as regular beam-separation scans. During both BI and regular vdM scans, the transverse bunch density distributions are also determined by simultaneously fitting the beam-separation dependence (“evolution”) on the luminosity and the luminous region position, orientation, and spatial width, as reflected in the reconstructed primary vertices (as discussed in Sect. 4.4.2).

The LHC conditions at IP 5 for the luminosity calibration fills discussed in this paper for 2015 and 2016 are summarized in Table 1.

**Table 1:** Summary of the LHC conditions at IP 5 for the scan sessions in ${\text {p}}{\text {p}}$ collisions in 2015 and 2016. The column labeled $\mu$ is the average pileup corresponding to $\mathcal {L} _{\mathrm {init}}$, the latter denoting the initial instantaneous luminosity. The columns corresponding to “No. of scans” indicate the total number of vdM, BI, and LSC scans that were performed in either transverse coordinate, counting only scans used for analysis

Fill	$\sqrt{s}$ ($\text {TeV}$ )	Date	$n_{\mathrm {b}}$	$\phi$ ($\upmu$rad)	$\beta ^{*}$ (cm)	$\mu$	$\mathcal {L} _{\mathrm {init}}$ ($\times \text {10}^\text {30} \,\text {cm}^{-2}\,\text {s}^{-1}$)	No. of scans		
								vdM	BI	LSC
4266	13	Aug. 2015	30	0	1917	0.6	2.7	6	4	3
4945	13	May 2016	32	0	1917	0.6	2.5	–	–	2
4954	13	May 2016	32	0	1917	0.6	2.5	6	4	2

**Fig. 4:**
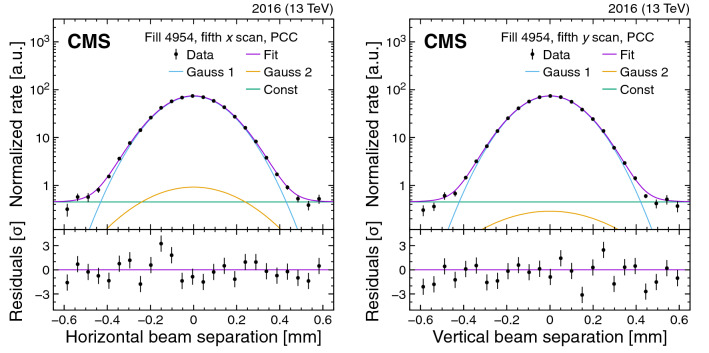
Example vdM scans for PCC for BCID 41, from the last scan pair in fill 4954, showing the rate normalized by the product of beam currents and its statistical uncertainty as a function of the beam separation in the *x* (left) and *y* (right) direction, and the fitted curves. The purple curve shows the overall double-Gaussian fit, while the blue, yellow, and green curves show the first and second Gaussian components and the constant component, respectively. All corrections described in Sect. 4.3 are applied. The lower panels display the difference between the measured and fitted values divided by the statistical uncertainty

**Fig. 5:**
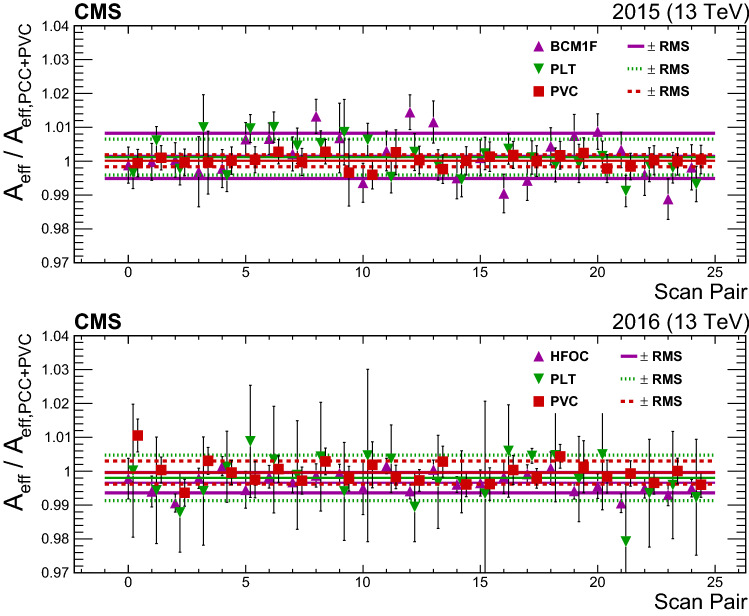
The two figures show comparisons of effective area ($A_{\mathrm {eff}}$) of cross-check luminometers with respect to the nominal PCC+PVC for fills 4266 (upper) and 4954 (lower). The points are the ratio of the $A_{\mathrm {eff}}$ of the labeled luminometer to PCC+PVC. There are 25 $A_{\mathrm {eff}}$ values because there are five scan pairs with five BCIDs analyzed for each scan pair. The solid lines are the average of all the $A_{\mathrm {eff}}$ while the bands are the standard deviations. In both sets of data the average comparison is compatible with unity within or near the standard deviation

Pixel data are collected for PCC and for methods involving collision vertices using a zero-bias trigger, which collects data from five BCIDs with a total rate of approximately 20$\text { kHz}$. Figure 4 shows vdM scan data from PCC recorded in the fifth scan pair of the session in fill 4954. The fit function corresponds to the double-Gaussian formalism of Eq. (14), and the parameters are estimated by simultaneously fitting the PCC and PVC rate measurements. An additional constant term is included to estimate the background originating from noncollision sources. This function provides a good description of the data in a range that extends over nearly three orders of magnitude in rate ($\chi ^2/\text {dof}\approx 1$ in Fig. 4). For other luminometers, background rates are either negligible (PLT and PVC) or estimated and subtracted (BCM1F and HFOC) prior to the beam parameter fit. Since the instantaneous luminosity is relatively low, any nonlinear effect has a negligibly small impact in any method. The beam-width parameters (Eq. (15)) measured using different luminometers are in excellent agreement, which is shown in Fig. 5 with comparisons of $A_{\mathrm {eff}}$ with the nominal PCC+PVC results.

**Fig. 6:**
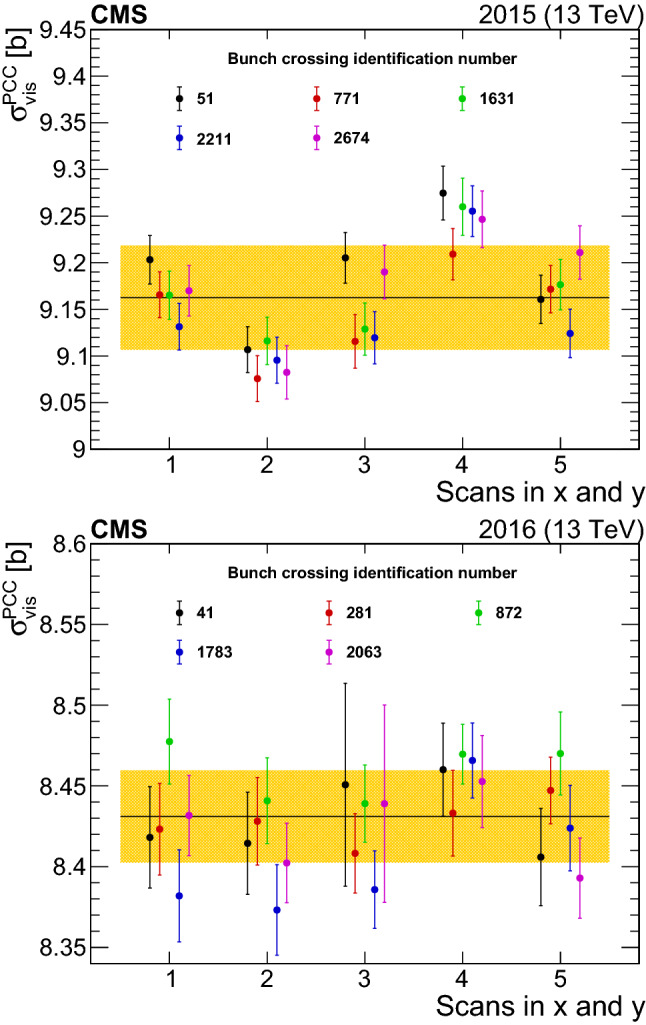
The measured $\sigma _{\mathrm {vis}}^{\text {PCC}}$, corrected for all the effects described in Sect. 4.3, shown chronologically for all vdM scan pairs (where 3 and 4 are BI scans) taken in fills 4266 (upper) and 4954 (lower), respectively. Each of the five colliding bunch pairs is marked with a different color. The error bars correspond to the statistical uncertainty propagated from the vdM fit to $\sigma _{\mathrm {vis}}^{\text {PCC}}$. The band is the standard deviation of all fitted $\sigma _{\mathrm {vis}}^{\text {PCC}}$ values

Although the accelerator parameters, such as bunch transverse sizes or intensities, vary during the course of a fill, such changes cancel in the calculation of $\sigma _{\mathrm {vis}}$, which should remain invariant. This is shown in Fig. 6 for the measured $\sigma _{\mathrm {vis}}^{\text {PCC}}$ as a function of time for vdM scans taken in fills 4266 and 4954. After including all the effects described in Sect. 4.3, $\sigma _{\mathrm {vis}}^{\text {PCC}} =9.166\pm 0.056\,\text {(stat)}$ and $8.429\pm 0.029\,\text {(stat)} \text { barns}$ in 2015 and 2016, respectively, where the bunch-by-bunch fit uncertainty in $\varSigma _x$, $\varSigma _y$, and $\mu _{\mathrm {vis}}$ is propagated to the measured $\sigma _{\mathrm {vis}}^{\text {PCC}}$ per scan. Since these uncertainties are statistical in nature, they contribute to the scan-to-scan combination in an uncorrelated way. The assumption of factorizable proton bunch densities limits the level of accuracy in the luminosity scale inferred from Eq. (13). A common approach is thus adopted at the LHC that includes a dedicated tailoring of the proton bunch injection chain to minimize the emergence of non-Gaussian bunch density distributions [43]. Since the factorizability between the *x* and *y* distributions could impact the vdM scan result of the different IPs differently, CMS reconstructs the individual proton bunch densities during the BI and vdM scans, as described in Sect. 4.4.

### Corrections to vdM scan data

Several systematic effects can change the measurement of $\sigma _{\mathrm {vis}}$, and the following sections describe the measurement of these effects, the corrections used, and the resulting systematic uncertainty in $\sigma _{\mathrm {vis}}$.

Adjustments to the bunch-by-bunch charge measurement are made to correct for spurious charge that is present outside the nominally filled part of the slot (Sect. 4.3.1). Then, we correct for potential sources of bias associated with the beam position monitoring at the scale of $\upmu$m. We distinguish between “orbit drifts”, which we model with smooth, linear functions, and residual differences relative to the nominal beam positions, where corrections per scan step are assessed. Since both effects are time dependent, thereby biasing $\sigma _{\mathrm {vis}}$ incoherently, they are monitored continuously during each scan (Sect. 4.3.2).

Another source of correction originates from the electromagnetic interaction between charged particles in the colliding bunches (beam-beam effects); when the beams are displaced, rather than being head-on, a beam deflection and change in $\beta ^{*}$ may be induced. The former causes the beams to be more separated than the nominal value from LHC beam position estimates, whereas the latter influences the spatial distributions of proton bunches and thus the observed rate. The resulting corrections to $\sigma _{\mathrm {vis}}$ are evaluated at IP 5 [44, 45], and depend on the LHC optics, beam parameters, and filling scheme (as discussed in Sect. 4.3.3).

The vdM method requires an accurate knowledge of the beam separation. Possible differences in the absolute scale between the nominal beam separation produced by the steering of the LHC magnets, as used in Eqs. (11) and (12), and the actual separation are determined by using the LSC procedure (Sect. 4.3.4).

#### Beam current calibration and spurious charge

The LHC beam currents are measured by dedicated devices. The FBCT system is used to measure the current of individual bunches in 25$\text { ns}$ bunch slots. The DCCT system provides a precise (0.2%) measurement of the total current for each of the two beams; since it is more precise than the FBCT sum, its scale is used to normalize the sum of the FBCT measurements.

Both the DCCT and FBCT measurements are sensitive to additional charges outside the actual colliding bunch. These components must be measured and subtracted. The LHC radio frequency (RF) cavities operate at 400$\text { MHz}$, so a single 25$\text { ns}$ wide bunch slot contains ten 2.5$\text { ns}$ wide “RF buckets”. Only one RF bucket in a given bunch slot is filled with protons, and, in principle, the other nine RF buckets are empty. Similarly, of the total 3564 bunch slots, only a predefined subset is filled, according to the filling scheme. In practice, however, a small amount of spurious charge is present in the nominally empty RF buckets and bunch slots, which should be subtracted from the $n_1$ and $n_2$ values in Eq. (13). The amount of “ghost” charge in the nominally empty bunch slots is included in the DCCT but not in the FBCT measurement, since the latter is insensitive to bunch charges below a certain threshold. The out-of-time (satellite) charge occupies RF buckets adjacent to the main bunch. As such, it can experience long-range interactions with the main bunch in the other beam and is visible in the FBCT measurement. The corrected value for $n^j$ (where *j* denotes the BCID) is therefore given by:

18 $$\begin{aligned} n^j = \frac{n^j_{\mathrm {FBCT}}\left( 1 - f^j_{\mathrm {sat}}\right) }{ \sum _j n^j_{\mathrm {FBCT}}} N_{\mathrm {DCCT}} \left( 1 - f_{\mathrm {ghost}}\right) , \end{aligned}$$

where $f^j_{\mathrm {sat}}$ represents the per-bunch correction due to the satellite bunch population and $f_{\mathrm {ghost}}$ is the correction for the ghost charge.

The spurious charge is measured by the LHC LDM system, which provides a precise longitudinal distribution of the beam charge with a time resolution of 90$\text { ps}$. The data from the LDMs for fills 4266 and 4954 indicate that both the ghost and satellite charges are small. The latter is estimated to be $< 0.1\%$ for each of the two beams and is neglected. No particular time dependence for either beam is observed, and the resulting overall spurious-charge correction in $\sigma _{\mathrm {vis}}$ amounts to $+$0.2 and $+$0.3% in 2015 and 2016, respectively. This is applied as a correction to the beam currents in Eq. (16).

The ghost charge is also measured using the beam-gas imaging method [12, 46, 47], which compares the beam-gas rates in bunch crossings at IP 8 (the location of the LHCb detector) where only one beam contains protons, or where neither beam contains protons, leading to consistent results with the LDM measurement. The systematic uncertainty of 0.1% is assigned to cover the difference between the two estimates of the ghost contributions to the beam current.

#### Beam position monitoring

Although the LHC beam orbits are generally stable during a fill, even a small variation (either random or systematic in nature) in the beam positions during scans can significantly affect the resulting calibrations. The beam positions are measured primarily using the DOROS BPM system. The LHC arc BPMs, when possible, are used to confirm the stability of the orbits during the scan.

**Fig. 7:**
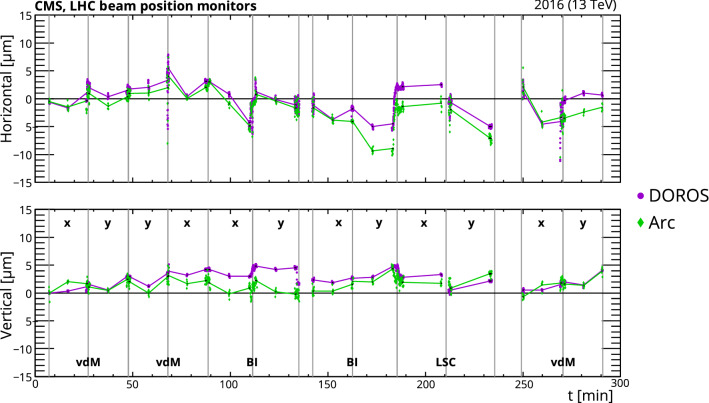
Effect of orbit drift in the horizontal (upper) and vertical (lower) beam-separation directions during fill 4954. The dots correspond to the beam positions measured by the DOROS or LHC arc BPMs in $\upmu \hbox {m}$ at times when the beams nominally collide head-on and in three periods per scan (before, during, and after) represented by the vertical lines. First-order polynomial fits are subsequently made to the input from BPMs (dots) and are used to estimate the orbit drift at each scan step. Slow, linear orbit drifts are corrected exactly in this manner, and more discrete discontinuities are corrected on average

To measure the orbit drift, we use the beam position measurements in *x* and *y* in three 15$\text { s}$ periods when the beams are nominally colliding head-on: immediately before and after each scan, as well as at the middle point of the scan, where the beams are also head-on. For each scan, a fit using a first-order polynomial is performed from the point before the scan to the middle point, and it is used to derive the correction for the first half of the scan. Similarly, a fit from the middle to the point after the scan is used to correct the second half of the scan. Figure 7 shows the measured positions along with the resulting fits. In general, the orbit drift during the 2015 and 2016 vdM scans is less than about 5$\,\upmu \text {m}$ for most of the scans. However, in the third scan of both series, the orbit drift was significant enough to shift $\sigma _{\mathrm {vis}}$ by approximately $+$1.0%. The corrections are derived using the average of the two BPM systems, and the largest deviation of the correction from each individual system from the nominal correction is taken as the value of the systematic uncertainty due to orbit drift. This is typically 0.1–0.2 % overall.

**Fig. 8:**
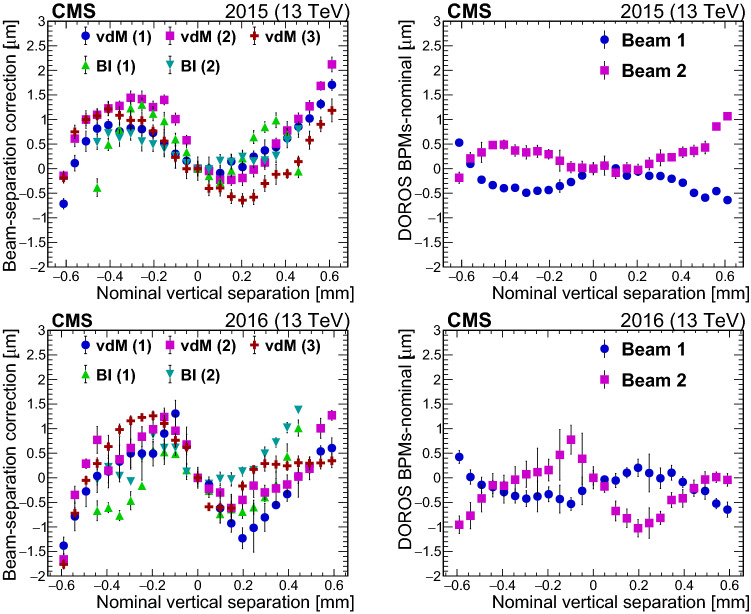
The beam-separation residuals in *y* during all scans in fills 4266 (upper) and 4954 (lower) are shown on the left. The dots correspond to the difference (in terms of beam separation in $\upmu$m) between the corrected beam positions measured by the DOROS BPMs and the beam separation provided by LHC magnets (“nominal”). The error bars denote the standard deviation in the measurements. The figures on the right show the residual position differences per beam between the DOROS BPMs and LHC positions for the first vdM scans in *y* in fills 4266 (upper) and 4954 (lower)

At each scan step, the actual beam separation can be also affected by systematic or random deviations of the beam positions from their nominal settings, which, in turn, impact the observed rate at each scan point. The magnitude of this potential bias is evaluated from consecutive single-beam orbit measurements at IP 5, provided by the DOROS BPMs and with a duration of a few seconds each. They are further corrected for the beam-beam effects (as discussed in Sect. 4.3.3) and the length scale (as described in Sect. 4.3.4) using the position of reconstructed vertices as the calibration target. The impact from beam-beam deflection at the location of the DOROS BPMs ($z_{\mathrm {DOROS}}=\pm 21.5\text { m}$ away from IP 5) is magnified by a factor of $1+\tan \left( \pi Q_{x/y}\right) z_{\mathrm {DOROS}}/\beta ^{*}$, where $Q_{x}$ and $Q_{y}$ are the betatron tune values in the *x* and *y* directions [33]. Because these values are different, the resulting factors are 2.7 in the *x* direction and 2.8 in the *y* direction. The measurements from the DOROS BPMs are integrated over all bunches. Therefore, the observed beam-beam deflection may be overestimated because of the inclusion of noncolliding, nondeflected bunches. In this analysis, a reduction factor of 0.6 is thus applied on top of the geometric factor in both years, which is the approximate fraction of the total number of bunches in the vdM fills that collide at IP 5. The orbit drift, as described above, is also subtracted from the single-beam DOROS measurements before forming the actual beam separation. Finally, an additional length scale correction is made to DOROS data for each beam and in both of the two transverse directions. The calibration using vertices, both for DOROS and nominal LHC positions, determines only the average length scale for the two beams. The calibrations of each beam are also not necessarily the same for the two sets of data. Therefore, a final, relative calibration of the DOROS data is made to align each beam in both transverse directions to the scale of the LHC beams. Figure 8 shows the residual difference in beam separation in all *y* scans in 2015 and 2016 as well as the residuals per beam in a single scan, which shows symmetric behavior. The resulting impact on $\sigma _{\mathrm {vis}}$ is in the range $-$0.6 to $+$0.4 and $-$0.5 to $-$0.2%, with average values of $-$0.1 and $-$0.3%, in 2015 and 2016, respectively. Corrections are applied for each scan, and the uncertainty comes from the reduction factor in the beam-beam deflection correction at the location of the DOROS BPMs.

#### Beam-beam effects

We distinguish two types of beam-beam interactions that affect the vdM and BI scan measurements: coherent and incoherent beam-beam effects. The total correction originates from the combination of both effects, which affect the nominal beam separation (coherent) and the detector rate (incoherent) via the change of the beam shapes.

**Fig. 9:**
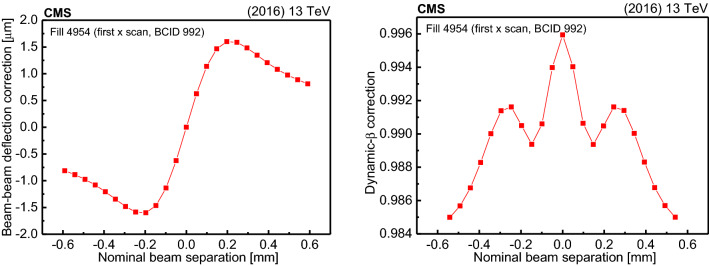
Calculated beam-beam deflection due to closed-orbit shift (left) and the multiplicative rate correction for PLT due to the dynamic-$\beta$ effect (right) as a function of the nominal beam separation for the beam parameters associated with fill 4954 (first scan, BCID 992). Lines represent first-order polynomial interpolations between any two adjacent values

The closed orbits of the bunches in the scans are shifted coherently by the angular kick induced by their electromagnetic repulsion, resulting in an increase in the absolute beam separation. The size of this additional beam-beam deflection depends on the transverse beam size, bunch intensities, collision optics, and separation between the orbits of colliding bunches. It is calculated based on the Bassetti–Erskine formalism for the electric field of elliptically distributed bunches, as discussed in Ref. [48]. The orbit shift depends linearly on the separation for small nominal beam separations, reaches a maximum near 2$\sigma _{\mathrm {b}}$ ($\approx 0.2\text { mm}$ in fills 4266 and 4954), and decreases nonlinearly towards zero at larger separations. Figure 9 (left) [44, 45] shows the resulting correction as a function of nominal beam separation, for the conditions during the scans in fill 4954 (Table 1). The beam-beam deflection correction increases the $\varSigma _x$ and $\varSigma _y$ values, impacting the $\sigma _{\mathrm {vis}}$ measurement by about $+$2.0 ($+$1.6)% in 2015 (2016).

The incoherent effect corresponds to the change of the proton bunch density distribution functions $\rho (x,y)$ at the IP due to deflection at the per-particle level. It causes a change in the effective $\beta ^{*}$, and thus results in a change in the measured luminosity. This dynamic evolution of $\beta ^{*}$ is usually referred to as the “dynamic-$\beta$” effect. The correction for the dynamic-$\beta$ effect is evaluated numerically by using a dedicated particle tracking program that calculates $A_{\mathrm {eff}}$ under different hypotheses [44, 45]. Considering the dynamic-$\beta$ effect independently of the beam-beam deflection, we obtain the ratio of the detector rate as shown in Fig. 9 (right). At vdM conditions, the dynamic-$\beta$ correction can be up to about $-2\%$ at large values of beam separation. Figure 9 shows the effect is typically larger at higher beam separation. In contrast to the beam-beam deflection, the dynamic-$\beta$ correction thus decreases the original $\varSigma _x$ and $\varSigma _y$ values. The corresponding impact on the calculated $\sigma _{\mathrm {vis}}$ is about −1.7 (−1.4)% in 2015 (2016).

The total beam-beam correction (i.e., when both the beam-beam deflection and dynamic-$\beta$ effects are included) results in an increase in the calculated $\sigma _{\mathrm {vis}}$ of about 0.3 (0.2)% in 2015 (2016) at IP 5. In addition, when considering further head-on collisions at the IP at the opposite side of the ring (IP 1 at ATLAS), the effect is approximated as a single-IP simulation but with shifted betatron tune values. The impact on $\sigma _{\mathrm {vis}}$ is enhanced by a factor of about two, leading to a total beam-beam correction of $+$0.6 ($+$0.4)% in 2015 (2016). The uncertainty in this calculation is dominated by the uncertainty in the betatron tune values, which was estimated taking into account the symmetric tune spread as well as the full shift due to head-on collisions at a second interaction point (in ATLAS at IP 1). These considerations translate into an uncertainty of 0.5 % in the corrected $\sigma _{\mathrm {vis}}$  [44, 45].

#### Length scale calibration

In the canonical vdM formalism described in Sect. 4.1, it is implicitly assumed that the beam separation is perfectly known. Operationally, the nominal displacement of the beams at the IP is achieved based on a local distortion (bump) of the orbit using a pair of steering dipoles located on either side of the IP [49]. The size of the nominal separation is subject to potential uncertainty associated with the response of the steering dipoles themselves (e.g., magnet hysteresis) or lattice imperfection [41], i.e., higher multipole components in the quadrupoles located within those orbit bumps. For a given IP, there are four possible bumps, for the two possible displacement directions of the two beams.

An accurate calibration for the size of the bumps can be obtained using the CMS tracker. In particular, for small vertex displacements, the uncertainty in the reconstructed vertex position in *x* or *y* is $\approx 20\,\upmu \text {m}$ for zero-bias collisions [29]. During LSC scans, the data for each separation distance contains several hundred thousand reconstructed vertices, yielding a position measurement with submicron precision.

The vdM scans described in Sect. 4.2 are typically done by moving the beams in equal steps in opposite directions. Since the two beams have independent length scales, the full separation correction is obtained from the mean of the length scale corrections per beam. Separate scans, wherein both beams are moved in steps in the same direction, are thus required to obtain the LSC. A more detailed description on the relationship between the calibration constant associated with the “offset” (i.e., the arithmetic mean between the transverse beam positions) and the observed quantities during LSC scans can be found in Ref. [12]. Here, for each scan step, the centroid of the luminous region is measured as the mean from a Gaussian fit to the observed vertex positions. A calibration constant for each transverse direction is extracted with a first-order polynomial fit to the difference between the measured mean position and the nominal offset as a function of the latter. This constant corresponds to the average calibration of the bumps of the two beams. It is then applied as a scale factor to correct the nominal beam displacement.

The nominal offset is also affected by the random and systematic beam position deviations described in Sect. 4.3.2. The beam positions at each step are monitored using DOROS BPMs. We estimate the arithmetic mean of the measured step sizes as a good representation of the nominal settings, after excluding outlier step sizes based on an iterative procedure. The difference of the remaining step sizes from the mean is used to correct the nominal offsets, and their standard deviation is the uncertainty due to beam position deviations. The correction improves the quality of the first-order polynomial fits and the forward-to-backward scan agreement. Consistent results are also found using the LHC arc BPMs to derive the correction for beam position deviations.

**Fig. 10:**
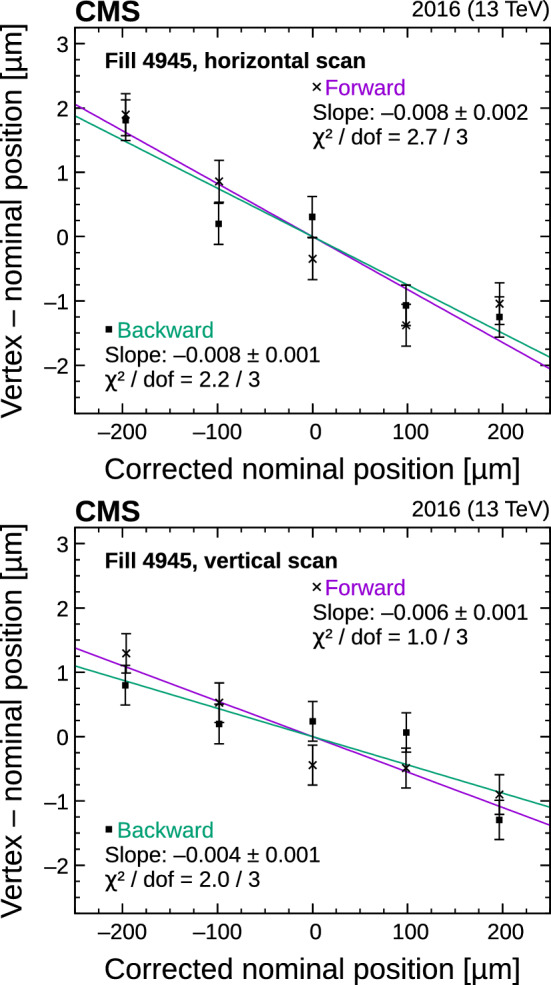
Fits to LSC forward (purple) and backward (green) scan data for the *x* (upper) and *y* (lower) LSC scans in fill 4945. The error bars denote the statistical uncertainty in the fitted luminous region centroid

The fit results for the *x* and *y* LSC scans are shown in Fig. 10 for fill 4945. The difference between the measured displacement of the beam centroid and the nominal displacement of the beams, corrected for the estimated beam position deviation, is plotted as a function of the latter. In all cases, the data are well described by first-order polynomial fits with calibration constants differing on average from zero by $-0.3$ and $-0.8\%$ in the horizontal plane in 2015 and 2016, respectively, and by $-0.1$ and $-0.5\%$ in the vertical plane. The combined correction to the visible cross section is $(-0.4 \pm 0.2)$ and $(-1.3 \pm 0.3)\%$. The total uncertainty, equal to the uncertainty contributions from the *x* and *y* planes added in quadrature, includes the statistical uncertainty in the first-order polynomial fits ($<0.1\%$), the variation between the two scan directions and the different scans (0.1%), a tracker alignment uncertainty ($<0.1\%$), and the uncertainty from the estimated beam position deviations (0.1–0.2%).

### Transverse factorizability

The use of the vdM scan technique to measure $A_{\mathrm {eff}}$ relies on the assumption that the proton bunch density functions are factorizable into *x*- and *y*-dependent components, as described in Sect. 4.1. If this condition is not met exactly, the measurements of $A_{\mathrm {eff}}$ and $\sigma _{\mathrm {vis}}$ will be biased. To correct for this potential bias, the bunch density distributions are measured independently with two methods, which are used in a combined way to evaluate $A_{\mathrm {eff}}$. In both methods, primary vertices are reconstructed from tracks measured in the CMS silicon tracker.

**Fig. 11:**
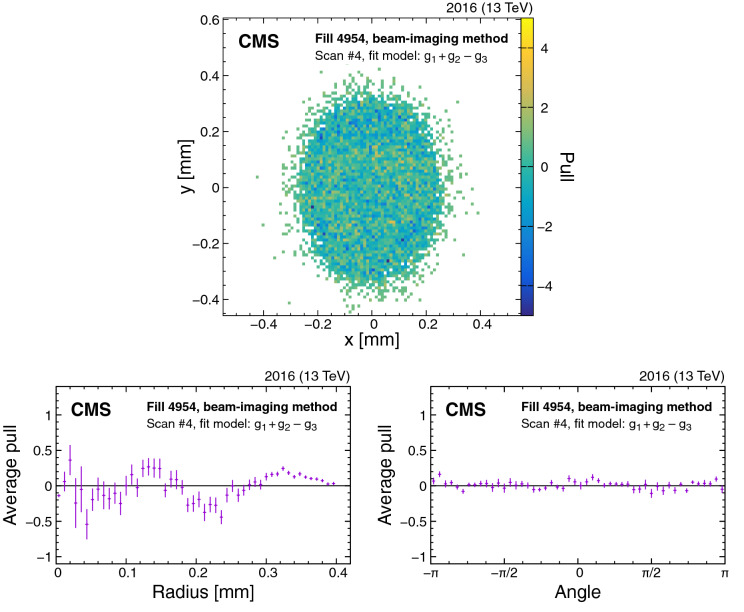
Example of the pull distributions of the fit model of Eq. (22) with respect to the vertex distribution that constrains beam 2 in the *y* direction recorded in fill 4954. The upper plot shows the two-dimensional pull distributions, and the lower plots show the per-bin pulls averaged over the same radial distance (lower left) or angle (lower right). The error bars in the lower plot denote the standard error in the mean of the pulls in each bin. The fluctuations observed in the radial projection of the residuals are included in the uncertainty estimation

#### Beam-imaging method

In the BI method [50, 51], the distributions of reconstructed vertices during BI scans are used to obtain an image of the transverse bunch profiles integrated over the scanning direction. A primary vertex resolution comparable to or smaller than the transverse beam sizes is necessary to extract the beam profiles from the measured distributions. The two-dimensional distribution in *x* and *y* of the reconstructed vertices depends on the overlap of the bunch density functions, their transverse separations $\varDelta x$ and $\varDelta y$, and the vertex resolution *V* of the CMS tracker system as:

19 $$\begin{aligned} N^{\text {vtx}}(x, y; \varDelta x, \varDelta y) \propto \rho _1(x,y) \rho _2(x+\varDelta x,y+\varDelta y) \otimes V. \end{aligned}$$

The combination of the vertex distributions from all steps of the BI scan in the *x* direction is approximated as:

20 $$\begin{aligned}&\sum _{\varDelta x=-4.5\sigma _b}^{+4.5\sigma _b} N^{\text {vtx}}(x, y;\varDelta x, \varDelta y)\nonumber \\&\quad \approx \bigg [\int \rho _1(x,y)\rho _2(x+\varDelta x,y) {\text {d}}(\varDelta x)\bigg ]\otimes V\nonumber \\&\quad = \rho _1(x,y) (\mathcal {M}_x \rho _2)(y) \otimes V. \end{aligned}$$

Here, $(\mathcal {M}_x \rho _2)(y)=\int \rho _2(x,y){\text {d}}x$ denotes that the proton bunch density of the second beam appears marginalized in the direction of the scan. This results from the assumption that the step size is small enough with respect to the width of the bunch densities, so we can replace the sum over discrete scan points with a continuous integral over $\varDelta x$. This two-dimensional vertex distribution can be exploited to constrain the transverse correlations of the bunch density of the first beam.

Combining four such vertex distributions accumulated during the BI scan set, we reconstruct the two-dimensional proton bunch densities of the two beams from a simultaneous fit. This requires knowledge of the primary vertex resolution, which is modeled with a two-dimensional Gaussian function. Convolving with the primary vertex resolution is then analytically possible for bunch density models built from Gaussian functions.

Models for the proton bunch density are built from Gaussian distributions parameterized with an additional correlation parameter $\varrho$:

21 $$\begin{aligned} g_j(x,y)= & {} \frac{1}{2\pi \sigma _{jx} \sigma _{jy} \sqrt{\smash [b]{1-\varrho _j^2}}} \exp {\Bigg (-\frac{1}{2(1-\varrho _j^2)}} \nonumber \\&\times \Bigg [ \frac{x^2}{\sigma _{jx}^2} +\frac{y^2}{\sigma _{jy}^2}-\frac{2\varrho _jxy}{\sigma _{jx}\sigma _{jy}}\Bigg ]\Bigg ), \end{aligned}$$

where *j* indicates the beam number ($j=1$ or 2). More complicated models are constructed with sums of these individual correlated Gaussian distributions. Distributions with a wide tail are better described by adding a Gaussian component with a small weight and a large width. Distributions with a flattened central part can be modeled with an additional component with a small negative weight and a narrow width. Typically, both nonzero correlation parameters and different widths are required to describe the nonfactorizability observed in data.

The best description of the BI data collected in 2015 and 2016 for the five bunch crossings used is achieved consistently with a sum of three Gaussian distributions, where the narrow component has a negative weight:

22 $$\begin{aligned} \rho _j (x,y)= & {} - w_{j,1} g_{j,1}(x,y) + w_{j,2} g_{j,2}(x,y) \nonumber \\&+ (1+w_{j,1}-w_{j,2}) g_{j,3}(x,y). \end{aligned}$$

Figure 11 shows the two-dimensional pull distribution, i.e., $(N^{\text {vtx}}_{\mathrm {data}}-N^{\text {vtx}}_{\mathrm {fit}})/\sigma _{\mathrm {data}}$, and the one-dimensional projections for the vertex distributions collected in the BI scan where the first beam is moved vertically for one bunch crossing in fill 4954. In these fits, the effects from the beam-beam deflection and dynamic-$\beta$ are included in the positions of the reconstructed vertices and as per-vertex weights, respectively, whereas the impact of orbit drift is negligibly small.

**Fig. 12:**
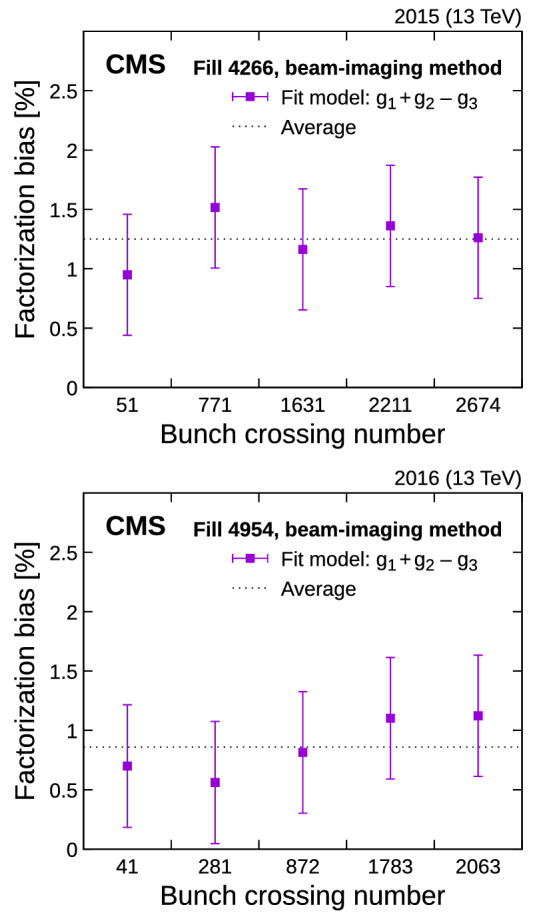
Factorization bias estimated from the fits to the BI bunch-by-bunch data in fills 4266 (upper) and 4954 (lower). The error bars denote sources of uncertainty (statistical and systematic), added in quadrature, in the factorization bias estimates

The value of $A_{\mathrm {eff}}$ can then be calculated from an integration of the overlap of the bunch densities directly (i.e., $A_{\mathrm {eff}} =\iint \rho _1(x,y)\rho _2(x,y){\text {d}}x{\text {d}}y$). This is compared to the value of $A_{\mathrm {eff}}$ obtained from an MC simulated vdM scan pair generated with the reconstructed bunch densities as input, and analyzed with the vdM method (i.e., $A_{\mathrm {eff}} =1/(2\pi \varSigma _x^{\text {MC}}\varSigma _y^{\text {MC}})$). The difference between the two values yields the bias of the vdM results, and is applied as a correction to $\sigma _{\mathrm {vis}}$ values. The bias is computed separately for each bunch crossing, and the results are shown in Fig. 12. The values for the estimated bias are averaged, resulting in a correction of $+$1.3 (0.9)% in $\sigma _{\mathrm {vis}}$ for 2015 (2016) because of the assumption of *x*-*y* factorization.

To estimate the uncertainty in the measured bias, the MC simulation of the vdM scans is repeated multiple times and the RMS of the resulting biases is 0.1% for both years, which is considered as the statistical uncertainty in the vdM scans. Additionally, a systematic uncertainty is evaluated with a closure test: simulated models are constructed by randomly drawing parameters of the fit model in Eq. (22). These are used to simulate MC pseudo-experiments by generating BI scan data, which are then fitted with the same model and procedure. Comparing simulated models with fit quality and fitted correction values similar to the data fits, the bias obtained from the bunch densities reconstructed from the fit agrees well on average with the true bias of the simulated model. The RMS of the distributions of deviations is 0.5 % for both years. We assign this RMS as the systematic uncertainty.

#### Luminous region evolution

In this method, which was inspired by Ref. [13], the luminosity and luminous region geometry are used to reconstruct the bunch density distributions in three dimensions and as a function of time. Using single-beam parameters, described in the following, bunch profiles are then generated for simulated vdM scans and treated as genuine vdM scan data. Similar to the BI method, the impact of factorization is extracted by comparing the “measured” luminosity extracted from the one-dimensional vdM simulated bunch profiles with the “true” luminosity from the computed four-dimensional (*x*, *y*, *z*, *t*) overlap integral of the single-bunch distributions. The luminous region is modeled by a three-dimensional ellipsoid whose parameters (nine in total) are extracted from an unbinned maximum likelihood fit of a three-dimensional Gaussian function to the spatial distribution of the primary vertices [29]. The vertex resolution is determined from data as part of the fitting procedure.

**Fig. 13:**
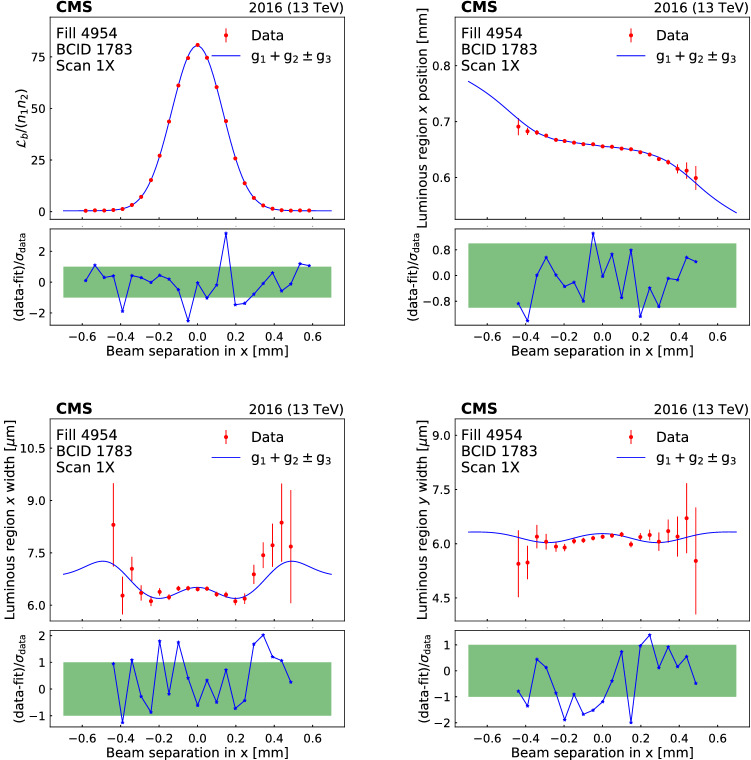
Beam-separation dependence of the luminosity and some luminous region parameters during the first horizontal vdM scan in fill 4954. The points represent the luminosity normalized by the beam current product (upper left), the horizontal position of the luminous centroid (upper right), and the horizontal and vertical luminous region widths (lower left and right). The error bars represent the statistical uncertainty in the luminosity, and the fit uncertainty in the luminous region parameters. The line is the result of the three-Gaussian ($g_1+g_2\pm g_3$) fit described in the text. In all cases, the lower panels show the one-dimensional pulls

The bunch profiles $\rho _j(x,y,z)$, parameterized per beam *j*, are the sum of three individual Gaussian distributions $g_{j,1\ldots 3}(x,y,z)$ with common mean, but arbitrary width and orientation parameters (referred to as “bunch parameters” in the following):

23 $$\begin{aligned} \rho _j(x,y,z)= & {} w_{j,1} g_{j,1}(x,y,z) + w_{j,2} g_{j,2}(x,y,z) \nonumber \\&+ (1-w_{j,1}-w_{j,2}) g_{j,3}(x,y,z). \end{aligned}$$

The overlap integral of Eq. (23) is evaluated at each scan step to predict the true luminosity and the geometry of the luminous region for a given set of bunch parameters. In this calculation, we consider the impact of beam-beam effects, LSC, and orbit drifts. The bunch parameters are then adjusted according to a $\chi ^2$ minimization procedure to determine the best-fit centroid position, orientation, and the widths (corrected for the primary vertex resolution) of the luminous region measured at each step of a BI or vdM scan. An example of a fit to the PCC luminosity and luminous region geometry is illustrated in Fig. 13 for one of the horizontal scans in fill 4954 and a subset of the three-dimensional ellipsoid parameters. One of the four figures shows the variation in the beam width in *y* during the *x*-separation beam scan, which is indicative of nonfactorization. The goodness of fit is better than $\chi ^2 / \text {dof} = 1.8$ for both years, with some systematic deviations being apparent mainly in the tails of the scan. The fits are repeated by substituting PLT as the luminosity input, but no particular dependence is seen.

**Fig. 14:**
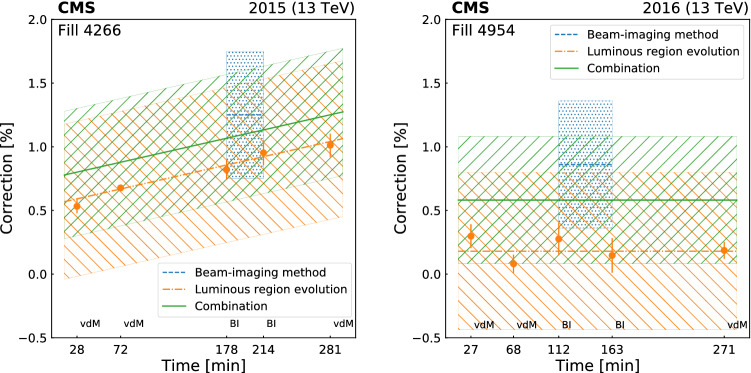
Ratio of the $\sigma _{\mathrm {vis}}$ evaluated from the overlap integral of the reconstructed single-bunch profiles in two (BI method) or three (luminous region evolution) spatial dimensions to that determined by the vdM method, assuming factorization, and their combination. The central values are displayed as points or with a line while the corresponding full uncertainties are shown as hatched areas. Different methods (including the combination) are color coded. Each point corresponds to one scan pair in fills 4266 (left) and 4954 (right). The statistical uncertainty is shown by the error bars

This procedure is applied to all (i.e., BI and vdM) scans in fills 4266 and 4954, and the results are summarized in Fig. 14. The $\sigma _{\mathrm {vis}}$ extracted from the standard vdM analysis with the assumption that factorization is valid is smaller by 0.6–1.1 (0.2)% than that computed from the reconstructed single-bunch parameters in fill 4266 (4954). Similar to the evaluation in the BI method, the uncertainty amounts to 0.6 %. This uncertainty is dominated by the standard deviation in simulation-driven closure tests, and includes the fit uncertainty in data and the contributions from beam-beam effects, length scale, and orbit drift. These observations are thus consistent with the ones obtained in Sect. 4.4.1 in terms of absolute magnitude during the BI scans. The two results are combined to produce the final correction in $\sigma _{\mathrm {vis}}$ of $+(0.8 \text {--}1.3 \pm 0.5)$ and $+(0.6 \pm 0.5)$% in 2015 and 2016, respectively. The final corrections retain the time evolution derived uniquely from the luminous region evolution method.

## Rate corrections under physics running conditions

The calibration scans described in the previous sections are performed with a small number of well-separated proton bunches with low bunch intensity. In contrast, during nominal conditions, the collision rate is generally maximized to produce large data sets for physics measurements and searches. This section describes the corrections that are applied to uncalibrated luminometer rates to ensure that the final luminosity values are accurate. These corrections, summarized in Table 2 for 2016, compensate for out-of-time pileup, efficiency, and nonlinearity effects for each individual luminometer.

**Table 2:** Summary of the rate corrections under physics running conditions in 2016 applied separately to each luminometer. For HFOC, two distinct sources of out-of-time pileup corrections are provided. In the first and second columns, the vdM calibration condition and the relative agreement of the luminometers in terms of $A_{\mathrm {eff}}$ relative to PCC during fill 4954 are given, respectively. The DT luminosity is also corrected for a very small additional muon rate from beam halo and cosmic sources, which is treated as a constant per fill

	vdM calibrated	vdM calibration agreement to PCC (%)	Out-of-time pileup corrections (%)	Efficiency corrections (%)	Nonlinear response (%)
PCC	Yes	–	0–4	1	–
DT	No	–	–	–	–
HFOC	Yes	0.2	0–15, 1–5	1	0–10
PLT	Yes	0.1	–	0–10	−0.2 to $+$1.4/(Hz/$\upmu$b)
PVC	Yes	<0.1	–	–	–
RAMSES	No	–	–	–	–

### Out-of-time pileup corrections

The measurements in most detectors have out-of-time pileup contributions that do not arise from the in-time ${\text {p}}{\text {p}}$ collision within the 25 ns window of the bunch crossing. Ideally, these contributions should be subtracted from all bunch crossings before the total instantaneous luminosity is computed. There are generally two types of effects that are considered: spillover of electronic signals and real additional response from material activation. These are denoted as type 1 (T1) and 2 (T2) afterglow, respectively.

The T1 afterglow generally only impacts the following bunch crossing because electronic signals tend to decline exponentially and hence two bunches later (50$\text { ns}$) the signal is again below threshold. The T1 contribution in bunch $n+1$ from bunch *n* is proportional to $\mathcal {L} _{\mathrm {b}} (n)$. Thus, the model for the correction is:

24 $$\begin{aligned} \mathcal {L} _{\mathrm {b,corr}}(n+1)=\mathcal {L} _{\mathrm {b,uncorr}}(n+1)-\alpha _{\mathrm {T1}} \mathcal {L} _{\mathrm {b,corr}}(n), \end{aligned}$$

where $\alpha _{\mathrm {T1}}$ is detector dependent and sometimes time dependent; $\alpha _{\mathrm {T1}}$ ranges from 0.005 for BCM1F to 0.02 for HFOC to as large as 0.09 for PCC.

In contrast, T2 afterglow tends to impact all bunch crossings, because the half-life of the activated material can be longer than several bunch crossings. The response can be modeled with a single- or double-exponential distribution. The impact of T2 afterglow varies by filling scheme and by detector. In fills where $n_{\mathrm {b}}$ is low and where the bunches are well separated, the T2 corrections are very small and often completely negligible, as is the case by design in the vdM calibration fills. When LHC fills contain several hundred bunches, the corrections start to contribute at the percent level in most bunches. With maximally full filling schemes, the corrections can be up to about 4 (15)% for PCC (HFOC).

Although there are clearly two distinct components, a combined (T1 and T2) model can be constructed that gives the response for a specific bunch crossing, accounting for contributions from all other 3563 bunch crossing slots. This model is referred to as the single-bunch response (SBR). The SBR for HFOC luminosity is taken directly from data in a reference fill with $n_{\mathrm {b}} =2$ for approximately the first half of the bunch crossings, and the bunches in the second half are smoothly extrapolated using an exponential model. The SBR is normalized to $\mathcal {L} _{\mathrm {b}} (n)$ and it is then subtracted from all other bunch slots. This procedure is repeated for all bunch crossings.

**Fig. 15:**
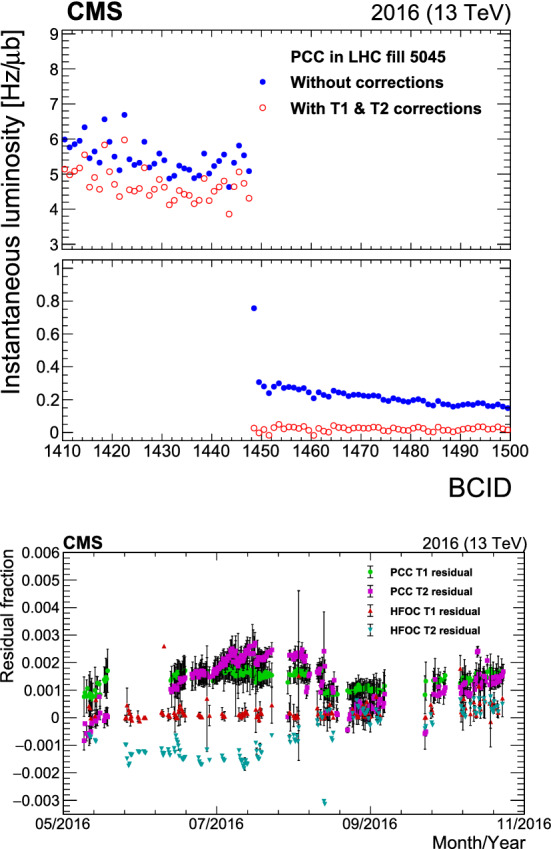
The upper plot shows the instantaneous luminosity measured from PCC as a function of BCID before (filled blue points) and after (open red points) afterglow corrections are applied for each colliding bunch. The upper panel shows a subset of bunch crossings colliding at IP 5, and the lower panel shows empty bunch crossings (the scale is different in the two panels to show differences more clearly). The open red points in the lower panel lie close to 0, indicating that any residual PCC response is small in empty bunch slots. The lower plot shows the estimated residual T1 and T2 afterglow as a function of time during the full range of 2016 data for both PCC and HFOC, which use the same afterglow subtraction methodology

After the corrections from the SBR are applied, empty bunch slots, where there are no collisions, should have a rate of zero. For PCC, the SBR is determined by optimizing $\alpha _{\mathrm {T1}}$, which is time dependent and measured in intervals of about 20$\text { min}$, and the parameters of the exponential used for T2 corrections, such that there is minimal residual rate in the noncolliding bunch slots. Figure 15 shows per-bunch data in a fill from 2016 before and after the afterglow corrections for PCC are applied.

The empty bunch slots are also used to estimate the residual afterglow after the full set of corrections is applied. The corrected rate in the first empty bunch slot after a colliding bunch slot is used to estimate the residual T1 response. Likewise, the 2nd to 30th empty bunch slots are used to estimate the residual T2 effect. This procedure is performed for the entire 2015 and 2016 data sets for PCC and HFOC luminosity measurements. A window covering all residuals over the course of each data set is used as the systematic uncertainty in the final corrections. The resulting uncertainty for PCC in the two corrections is $0.3 \bigoplus 0.1$ ($0.3 \bigoplus 0.3$)% in 2015 (2016).

These types of per-bunch luminosity corrections are applied for PCC, HFOC, and BCM1F, whereas PLT is almost completely background free and no such correction is needed. Since the DT and RAMSES measurements integrate over all bunch crossings, out-of-time pileup corrections can only be applied on average to the integrated rates. For DT these amount to 0–1%, while no corrections are applied to RAMSES.

A second type of T1 afterglow affects the HFOC luminosity. This is the case where the afterglow from a preceding bunch and the signal from the current bunch are both under the threshold to be counted as a hit, but their sum exceeds the threshold. This effect is referred to as the “bunch train effect”, because it affects only active bunches preceded by other active bunches (that is, bunches within a train, as opposed to “leading” bunches at the beginning of a train). The method previously described for estimating T1 afterglow does not include this contribution. This effect is measured in a dedicated study comparing the double ratio of the leading bunch in a train relative to the second bunch for HFOC divided by the same ratio for PCC. A single correction model with magnitude 1–5%, linearly increasing with instantaneous luminosity, is determined utilizing most valid data from 2016.

### Efficiency corrections

Radiation damage can affect the detector response by reducing efficiency, increasing noise, or both. Noise is typically a small effect for most luminometers, but reduced response in detectors due to radiation damage can have significant (percent-level) effects, and so corrections are required. Corrections are measured against a stable benchmark relative to the performance at or near the vdM scans, and are applied to $\sigma _{\mathrm {vis}}$. Shifts of 0–10% in detector response in the PLT in 2016 are corrected using RAMSES as a benchmark, whereas the impact of radiation damage on the HF efficiency is corrected using a parameterization derived from a model of aging. An HF efficiency correction of 1% is derived by measuring the average energy deposits in the HF in events characterized by the presences of ${\text {Z}}$bosons that decay to two muons with large transverse momentum.

A further efficiency correction is necessary for the PCC measurement. The pixel detector has a static internal memory buffer for data storage before the trigger decision is taken. When the buffer is filled, the oldest data overflows and are lost. This effect is proportional to the total instantaneous luminosity, and it can be estimated by studying the frequency of missing pixel clusters in otherwise well-reconstructed tracks [29]. In 2016, the effect was 1.0% at 1.4$\times \text {10}^\text {34}$ $\,\text {cm}^{-2}\,\text {s}^{-1}$. A correction proportional to total instantaneous luminosity is applied, and the total impact on integrated luminosity is 0.2%. Since the total luminosity in 2015 is substantially lower, no correction is applied. The PCC also has very small noise corrections.

### Nonlinear response

In the absence of out-of-time pileup, the PCC luminosity is expected to be linear, according to simulations, so no corrections are applied. Moreover, the ratios of PCC to both DT and RAMSES luminosity measurements are highly compatible as a function of the instantaneous luminosity without any corrections. The HFOC response in 2015 and 2016, on the other hand, exhibits significant nonlinearity compared to the other luminometers. The main source of nonlinearity is the uncalibrated ADC-to-charge conversion applied at the time of data taking. Data from fill 5416, which exhibit a wide range of instantaneous luminosity, are used to model the correction for HFOC with a fourth-order polynomial. This smooth function extrapolates to the $\sigma _{\mathrm {vis}}$ calibration at low pileup within uncertainty. This single model is used to correct the nonlinear behavior of HFOC (0–10% higher response when compared to PCC) throughout 2016.

As described in Sect. 3, nonlinearity corrections are also needed for PLT. The corrections are modeled with a first-order polynomial. The parameters are time dependent, because of changes in the PLT operating conditions during the course of 2015 and 2016. These corrections, amounting to −0.2 to $+$1.4/(Hz/$\upmu$b), are derived by comparing with RAMSES data in five different periods.

**Fig. 16:**
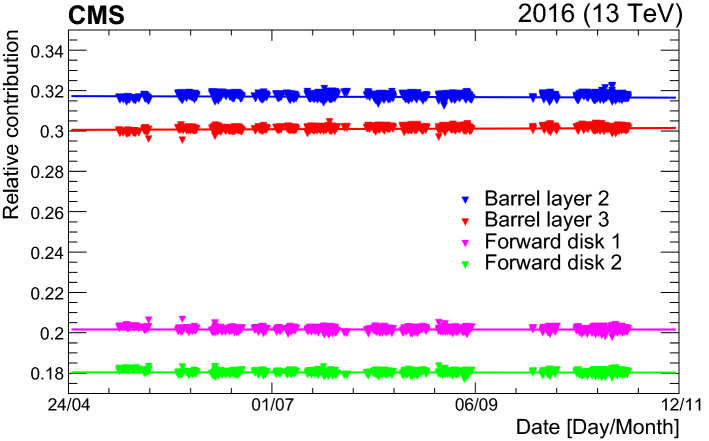
The relative contribution to the total number of observed pixel clusters from the four regions of the pixel detector used in the luminosity measurement (barrel layers 2 and 3, and inner and outer forward pixel disks), as a function of time throughout 2016. The lines represent first-order polynomial fits to the relative contributions from each region

## Detector stability and linearity

After the rate corrections are applied (as discussed in Sect. 5), comparisons between different luminometers are performed to assess remaining systematic effects impacting the luminosity measurement. Since PCC is expected to suffer the least from nonlinearities, as described in Sect. 2.1, once out-of-time pileup effects are corrected, PCC is the preferred luminometer for these data sets in the following estimates for stability and potential nonlinearity.

### Upper bounds on stability

One measurement of potential instability in the PCC luminosity comes from intrinsic monitoring (i.e., comparing rates from different sections/parts of the subdetector over time). In a perfectly stable system, the fractional rates among different subcomponents would exhibit no variation with time. Figure 16 shows the result of applying out-of-time pileup corrections (as discussed in Sect. 5.1) separately to each pixel layer or disk. The sum of the per-region corrections matches the total, nominal correction made for all PCC regions to better than 0.1%. After the corrections are applied, the relative rates are quite stable over the course of 2015 and 2016. This is also shown in Fig. 16, where the relative PCC rates over time are simultaneously fit to a first-order polynomial.

**Fig. 17:**
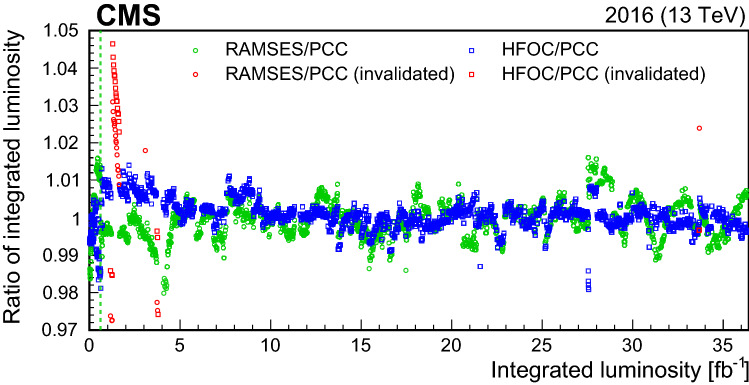
The luminosity measurements from PCC, HFOC, and RAMSES are compared as a function of the integrated luminosity in 2016. Comparison among three luminometers facilitates the identification of periods where a single luminometer suffers from transient stability issues. The ratios that are plotted in red contain invalidated data. The dashed line delineates the vdM calibration (fill 4954)

However, this method cannot detect global shifts in $\sigma _{\mathrm {vis}}$, and so it is crucial to make comparisons with completely independent systems. With multiple independent systems available for comparison, luminometers displaying brief periods of instability can be clearly identified. The cross-detector comparison is repeated for the entire data set for each year to detect periods where a single luminometer experiences transient effects (e.g., data quality issues, some detector components off, anomalous signals, etc.). Figure 17 shows the ratio of the luminosity measurements for different pairs of detectors throughout 2016, highlighting (in red) periods where the ratios significantly deviate from unity and so the associated data are invalidated.

**Fig. 18:**
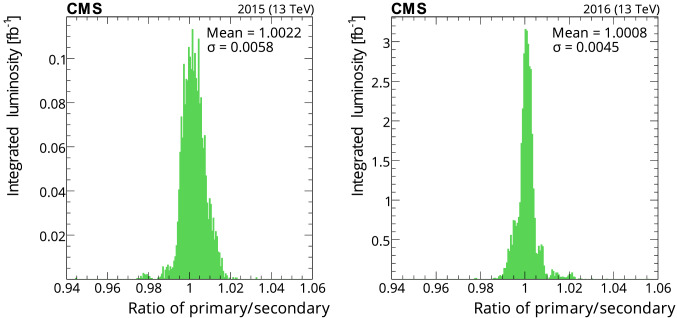
The ratio of the primary (best available) to secondary (next-best available) luminosity as computed in time windows of approximately 20$\text { min}$ each. The left plot shows the 2015 results (principally PCC/RAMSES), and the right plot shows the 2016 results (principally PCC/HFOC). Each entry is weighted by the integrated luminosity for the time period

After the exclusion of invalidated data, which amount to $\lesssim$5% for each luminometer, the remaining input from different luminometers is used to assess an upper limit on the stability of the luminosity. PCC measurements are valid for 98.3 (94.3)% of the data set in 2015 (2016). The rest of the luminosity is provided by the next most stable luminometer, which is RAMSES (HFOC) for 2015 (2016). The primary luminosity, which is PCC or luminosity from the next most stable detector when PCC is unavailable, is compared with the next-best available luminometer (secondary). In Fig. 18, the latter is selected using the lowest standard deviation in the ratio relative to PCC over fixed time intervals of approximately 20$\text { min}$ each. The position of the mean shows the agreement between the luminometers on the integrated luminosity. The width reflects stability effects, as well as residual statistical uncertainty in the luminosity measurement in each interval. From the distribution over the course of each year, the width is an upper limit on the uncertainty due to time dependencies in the luminometers. For 2015 (2016) a systematic uncertainty due to detector stability of 0.6 (0.5)% is derived.

### Time dependence of linearity

We make use of two methods for assessing the detector linearity. The primary method compares the ratio of the instantaneous luminosity from two luminometers per fill as a function of the instantaneous luminosity, which is estimated from the numerator. A first-order polynomial fit is performed and the slope is extracted. The slopes per fill are then studied as a function of time. No significant deviation over time is observed between DT/PCC or RAMSES/PCC and HFOC/PCC, DT/PCC, or RAMSES/PCC in 2015 and 2016, respectively.

**Fig. 19:**
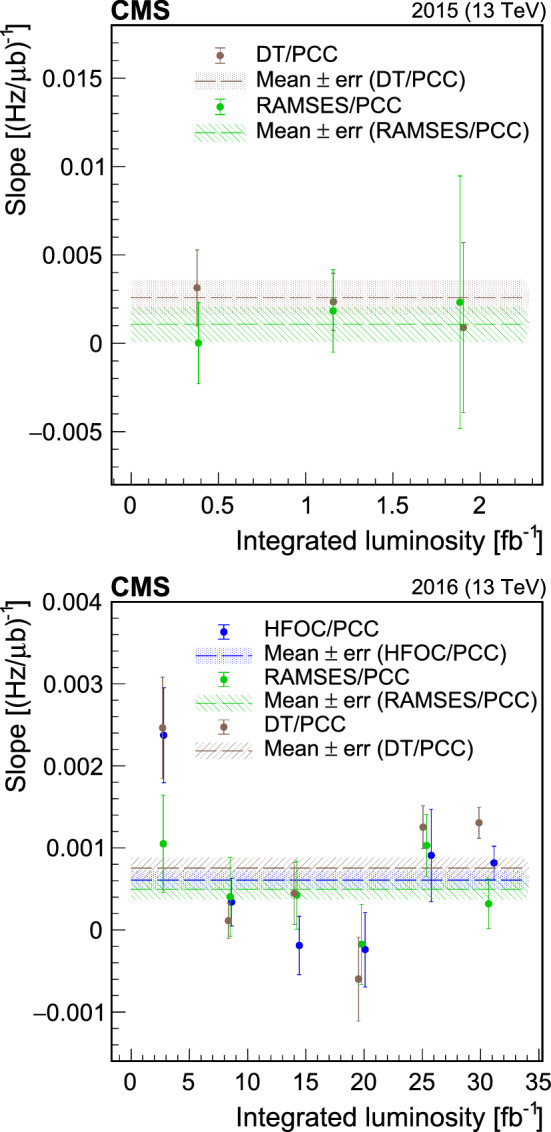
Linearity summary for 2015 (upper) and 2016 (lower) at $\sqrt{s}=13\,{\text {TeV}}$. The slopes are plotted for each detector relative to PCC. The markers are averages of fill-by-fill slopes from fits binned in roughly equal fractions of the total integrated luminosity through the year. The error bars on the markers are the propagated statistical uncertainty from fitted slope parameters in each fill, which are weighted by integrated luminosities of each fill. The dashed lines and corresponding hatched areas show the average from the entire data set and its uncertainty

To estimate the uncertainty, the fitted slopes are weighted according to the per-fill integrated luminosity. The mean values deviate slightly from 0, and the largest deviation is the systematic uncertainty in the linearity of PCC luminosity. Figure 19 shows the summary of these slopes for 2015 and 2016 at $\sqrt{s}=13\,{\text {TeV}}$ both for the whole year, and for subsets of each data set with equal luminosity. The largest average slope is 0.26 (0.08)%/($\hbox {Hz}/\upmu \hbox {b}$) in 2015 (2016), which translates into a 0.5 (0.3)% uncertainty in the integrated luminosity of the 2015 (2016) data set, where the average $\mathcal {L} _{\mathrm {b}}$ is approximately 2.0 (3.3) $\hbox {Hz}/\upmu \hbox {b}$.

The alternative method makes use of the entire data set throughout the year, and extracts a single relative slope with a first-order polynomial fit. To remove effects from variations in the absolute luminosity scale over time, the per-fill ratios are shifted such that their extrapolation at zero luminosity is unity. The results are consistent with the primary method described above.

## Total luminosity correction and uncertainty

For each data set, final rate corrections and final calibrations are applied to data in small time windows of $2^{18}$ LHC orbits, approximately 23 s. All the measurements are summed to derive a total integrated luminosity measurement. The contributions to the systematic uncertainty in the integrated luminosity are divided into two general categories:

- “normalization” uncertainty in the absolute luminosity scale, $\sigma _{\mathrm {vis}}$, determined from the vdM scan procedure
- “integration” uncertainty associated with $\sigma _{\mathrm {vis}}$ variations over time (stability) and pileup (linearity and out-of-time pileup corrections).

The magnitudes of the corrections applied to the absolute normalization from the vdM calibration are listed in Table 3, and Table 4 summarizes the sources of uncertainty. The dominant sources of normalization uncertainty are associated with the beam position monitoring (as discussed in Sect. 4.3.2), transverse factorizability (as explained in Sect. 4.4), and beam-beam effects (as described in Sect. 4.3.3).

The dominant sources of integration uncertainty arise from the linearity and stability of the primary relative to secondary luminosity measurements over the course of each year (as discussed in Sect. 6). In addition, the subleading systematic uncertainty due to out-of-time pileup corrections is considered for the PCC method since it is primarily used for the luminosity estimate.

Several sources of normalization uncertainty are considered to be correlated for the years studied because the scan procedures and analysis methodology are identical between the two vdM calibrations. The sources of the normalization uncertainty that are not correlated between the two vdM programs, and are partly statistical in nature, are the orbit drift, along with the scan-to-scan and bunch-to-bunch variations in the measured $\sigma _{\mathrm {vis}}$. The latter are collectively referred to as “other variations in $\sigma _{\mathrm {vis}}$ ” in Table 4.

Among the sources of integration uncertainty, the afterglow corrections are treated identically in the two data sets, and so this source of systematic uncertainty is correlated. The estimate of the uncertainty due to linearity is considered to be correlated, since it is derived from the PCC linearity in both years. On the other hand, the stability assessment is based on cross-detector comparisons. Although PCC is the primary luminometer in each data set, the secondary luminometer is different for each year. Since the source of instability cannot be assessed and contains time-dependent features, the uncertainty is not correlated.

The tool used for providing luminosity values to physics analyses applies the corrections to the raw luminosity values using the average per-bunch luminosity, rather than the individual bunch-by-bunch values. This potentially introduces an error in the case where these corrections include a nonlinear term and the bunch-by-bunch luminosity varies significantly among bunches. We evaluated the effect of this approximation on 2016 data, and found that the overall impact on the integrated luminosity was <0.1%.

Finally, the quantity measured by the luminometers is the luminosity delivered to CMS; however, the quantity of interest to most physics analyses is the luminosity corresponding to the data actually recorded by the CMS DAQ system. These are related by the deadtime, as obtained from the trigger and clock system of CMS [21]. In 2015 this measurement was affected by an algorithm issue in the trigger system and has an uncertainty of 0.5%, but this problem was resolved before data taking began in 2016, so in 2016 the impact is negligible (<0.1%) and uncorrelated with 2015.

When applying the vdM calibration to the entire periods, the total integrated luminosity is 2.27 $\,\text {fb}^{-1}$ with a relative precision of 1.6% in 2015, and 36.3 $\,\text {fb}^{-1}$ with a relative precision of 1.2% in 2016. The combined $2015+2016$ luminosity measurement has a precision of 1.2%, which is the same as the 2016 precision since it is the significantly larger data set and the precision in 2015 is similar.

**Table 3:** Summary of the BCID-averaged corrections to $\sigma _{\mathrm {vis}}$ (in %) obtained with the vdM scan calibrations at $\sqrt{s}=13\,{\text {TeV}}$ in 2015 and 2016. When a range is shown, it is because of possible scan-to-scan variations. To obtain the impact on $\sigma _{\mathrm {vis}}$, each correction is consecutively included, the fits are redone following the order below, and the result is compared with the baseline. The impact from transverse factorizability is obtained separately (as discussed in Sect. 4.4)

Source	Impact on $\sigma _{\mathrm {vis}}$ (%)	
	2015	2016
Ghost and satellite charge	$+$0.2	$+$0.3
Orbit drift	$+$0.6 to $+$1.0	$+$0.2 to $+$1.0
Residual beam position corrections	$-$0.6 to $+$0.4	$-$0.5 to $-$0.2
Beam-beam effects	$+$0.6	$+$0.4
Length scale calibration	−0.4	−1.3
Transverse factorizability	$+$0.8 to $+$1.3	$+$0.6

## Summary

The luminosity calibration using beam-separation (van der Meer, vdM) scans has been presented for data from proton–proton collisions recorded by the CMS experiment in 2015 and 2016 when all subdetectors were fully operational. The main sources of systematic uncertainty are related to residual differences between the measured beam positions and the ones provided by the operational settings of the LHC magnets, the factorizability of the transverse spatial distributions of proton bunches, and the modeling of effects on the proton distributions due to electromagnetic interactions among protons in the colliding bunches. When applying the vdM calibration to the entire data-taking period, the relative stability and linearity of luminosity subdetectors (luminometers) are included in the uncertainty in the integrated luminosity measurement as well.

The resulting relative precision in the calibration from the vdM scans is 1.3 (1.0)% in 2015 (2016) at $\sqrt{s}=13\,{\text {TeV}}$; the integration uncertainty due to luminometer-specific effects contributes 1.0 (0.7)%, resulting in a total uncertainty of 1.6   (1.2)%; when applying the vdM calibration to the entire periods, the total integrated luminosity is 2.27 (36.3)$\,\text {fb}^{-1}$.

**Table 4:** Summary of contributions to the relative systematic uncertainty in $\sigma _{\mathrm {vis}}$ (in %) at $\sqrt{s}=13\,{\text {TeV}}$ in 2015 and 2016. The systematic uncertainty is divided into groups affecting the description of the vdM profile and the bunch population product measurement (normalization), and the measurement of the rate in physics running conditions (integration). The fourth column indicates whether the sources of uncertainty are correlated between the two calibrations at $\sqrt{s}=13\,{\text {TeV}}$

Source	2015 (%)	2016 (%)	Corr
Normalization uncertainty			
*Bunch population*			
Ghost and satellite charge	0.1	0.1	Yes
Beam current normalization	0.2	0.2	Yes
*Beam position monitoring*			
Orbit drift	0.2	0.1	No
Residual differences	0.8	0.5	Yes
*Beam overlap description*			
Beam-beam effects	0.5	0.5	Yes
Length scale calibration	0.2	0.3	Yes
Transverse factorizability	0.5	0.5	Yes
*Result consistency*			
Other variations in $\sigma _{\mathrm {vis}}$	0.6	0.3	No
Integration uncertainty			
*Out-of-time pileup corrections*			
Type 1 corrections	0.3	0.3	Yes
Type 2 corrections	0.1	0.3	Yes
*Detector performance*			
Cross-detector stability	0.6	0.5	No
Linearity	0.5	0.3	Yes
*Data acquisition*			
CMS deadtime	0.5	< 0.1	No
Total normalization uncertainty	1.3	1.0	–
Total integration uncertainty	1.0	0.7	–
Total uncertainty	1.6	1.2	–

The final precision is among the best achieved at bunched-beam hadron colliders. Advanced techniques are used to estimate and correct for the bias associated with the beam position monitoring at the scale of $\upmu \hbox {m}$, the factorizability of the transverse beam distribution, and beam-beam effects. In addition, detailed luminometer rate corrections and the inclusion of novel measurements (such as the data from the Radiation Monitoring System for the Environment and Safety) lead to precise estimates of the stability and linearity over time.

In the coming years, a similarly precise calibration of the real-time luminosity delivered to the LHC will become increasingly important for standard operations. Under those conditions, the impact of out-of-time pileup effects is expected to be larger, but in principle they can be mitigated using techniques described in this paper.

## Data Availability

This manuscript has no associated data or the data will not be deposited. [Authors’ comment: Release and preservation of data used by the CMS Collaboration as the basis for publications is guided by the CMS policy as written in its document “CMS data preservation, re-use and open access policy” (https://cms-docdb.cern.ch/cgi-bin/PublicDocDB/RetrieveFile?docid=6032&filename=CMSDataPolicyV1.2.pdf&version=2).]
